# Infants’ looking preferences for social versus non-social objects reflect genetic variation

**DOI:** 10.1038/s41562-023-01764-w

**Published:** 2023-11-27

**Authors:** Ana Maria Portugal, Charlotte Viktorsson, Mark J. Taylor, Luke Mason, Kristiina Tammimies, Angelica Ronald, Terje Falck-Ytter

**Affiliations:** 1 Development and Neurodiversity Lab (DIVE), Department of Psychology, Uppsala University, Uppsala, Sweden; 2 Center of Neurodevelopmental Disorders (KIND), Centre for Psychiatry Research, Department of Women’s and Childrn’s Health, Karolinska Institutet & Stockholm Health Care Services, Stockholm, Sweden; 3 Department of Medical Epidemiology and Biostatistics, Karolinska Institutet, Stockholm, Sweden; 4 Department of Forensic and Neurodevelopmental Sciences, Institute of Psychiatry, Psychology and Neuroscience, King’s College London, London, UK; 5 Astrid Lindgren Children’s Hospital, Karolinska University Hospital, Stockholm, Sweden; 6 School of Psychology, Faculty of Health and Medical Sciences, University of Surrey, Guildford, UK; 7 Swedish Collegium for Advanced Study, Uppsala, Sweden

**Keywords:** Human behaviour, Attention, Behavioural genetics, Oculomotor system, Social neuroscience

## Abstract

To what extent do individual differences in infants’ early preference for faces versus non-facial objects reflect genetic and environmental factors? Here in a sample of 536 5-month-old same-sex twins, we assessed attention to faces using eye tracking in two ways: initial orienting to faces at the start of the trial (thought to reflect subcortical processing) and sustained face preference throughout the trial (thought to reflect emerging attention control). Twin model fitting suggested an influence of genetic and unique environmental effects, but there was no evidence for an effect of shared environment. The heritability of face orienting and preference were 0.19 (95% confidence interval (CI) 0.04 to 0.33) and 0.46 (95% CI 0.33 to 0.57), respectively. Face preference was associated positively with later parent-reported verbal competence (*β* = 0.14, 95% CI 0.03 to 0.25, *P* = 0.014, *R*^2^ = 0.018, *N* = 420). This study suggests that individual differences in young infants’ selection of perceptual input—social versus non-social—are heritable, providing a developmental perspective on gene–environment interplay occurring at the level of eye movements.

## Abstract (web summary)

This eye-tracking study of ~500 5-month-old infant twins indicates that individual preferences for faces versus non-social objects like cars and phones are associated with genetic variation.

## Main

From looking and interacting with other people, infants get experiences that contribute to shaping social brain circuits and social cognition. At the same time, the developing infant is confronted with the massive task of learning about non-social objects and events. Whether an infant looks at faces or at non-social objects at any moment in time can reflect both bottom-up and top-down processes, including interests, understanding and motivation, and the maturation of the cognitive system^1,2^. Atypical attention to social versus non-social objects has been implicated in autism, a heritable neurodevelopmental condition partly defined by social communication difficulties^3–6^. However, also in the typical population, there is substantial variation with regard to social versus non-social visual preferences^5,7,8^, and recent data suggest that specific aspects of social preferences such as attention to eyes versus mouth of other people’s faces are highly heritable in infants^9^ and young children^10^. The current study evaluated the extent to which visual preference for faces versus non-social information in early infancy reflects genetic variation in the population (which, at the extreme, could be associated with heritable clinical conditions such as autism^5,11,12^).

Faces selectively attract attention already at birth and act as a catalyst for cognitive, social and emotional development^13–16^. An early bias to orient to faces (fast first looks at face-like configurations in the periphery) is proposed to be subcortically mediated and present at birth, whereas sustained looking at faces requires later maturing cortical top-down structures^15^. By 6 months of age, top-down control enables flexible looking behaviour permitting the infant to preferentially attend to the face but also to shift attention away from it and to the other stimuli in the environment^15,17,18^.

In this Article, based on a pre-registered analysis plan^19^, we studied the genetic and environmental influences underlying individual differences in two early emerging aspects of selective attention to social versus non-social information: orienting (looking first at faces rather than non-social objects), and sustained preference (ratio of looking time in the face relative to other objects). We also assessed the infants’ efficiency of visual exploration, defined as how many of the objects in the stimulus array (social and non-social) infants looked at during the first 10 s following stimulus onset. We expected that variation in all three phenotypes studied would have a significant genetic component^9,10,20^. We also assessed the aetiological link between the eye-tracking phenotypes, but due to lack of previous research we had no specific hypotheses. Next, given the links reported between visual attention to faces and autism^3–6^ and between attention control and attention-deficit/hyperactivity disorder (ADHD)^11^, we also tested whether the different emerging aspects of attention to faces early in life were associated with polygenic scores (common genetic variance) and later traits related to autism and ADHD; specifically, we expected face preference at 5 months to be related to higher social communication abilities and the efficiency of visual exploration to be related to later self-regulation. Finally, because greater attention to faces in infancy is thought to predict better language outcomes later in life (for example, refs. ^21,22^), we also studied whether the different aspects of attention to faces were associated with later language skills, specifically we expected that face preference at 5 months would be related to higher language abilities. Data came from the Babytwins Study Sweden (BATSS)^23^, a Swedish community sample of dizygotic and monozygotic 5-month-old twins who went through gaze-based experimental measurements of looking at faces presented together with other non-face objects in a five-item array.

We used a classical twin modelling approach, in which one compares the level of within-pair similarity separately for monozygotic twins (MZ; who share 100% of their segregating genetic material) and dizygotic twins (DZ; who on average share 50%). Univariate twin models estimate the relative contribution of genetic and environmental factors to the variation in a phenotype, by comparing the correlation between twins; while bivariate twin models further estimate the relative contributions of genetic and environmental factors to the covariation between two phenotypes, by comparing cross-trait cross-twin (CTCT) correlations, that is, the correlation between one phenotype for one twin and another phenotype for their co-twin. The variation or covariation can be decomposed into additive genetic influences (A; heritability, which increases twin similarity), non-shared environment (E; environmental influences that differ between twins and decrease twin similarity, including measurement error), and shared environment (C; environmental influences that increase twin similarity regardless of zygosity, for example, family socioeconomic status).

## Results

**Fig. 1:**
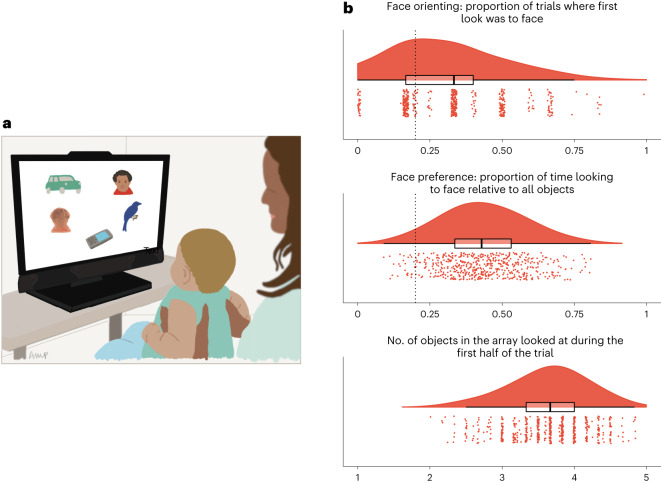
The face pop-out paradigm—illustrative set-up and primary looking measures data plots. **a**, An infant viewing one trial of the face pop-out task. Illustration by author A. M. P. **b**, Raincloud plots^54^ (centre lines represent the median, box limits represent upper and lower quartile, whiskers represent 1.5× interquartile range, and outliers are not presented) of the three primary looking measures derived from the task, across 536 5-month-old infants: face orienting (mean was significantly above chance level, highlighted as a dashed vertical line; one-sample two-tailed *V*_Twin 1_(273) = 25,558, *P* < 0.001, *d* = 0.54, 95% CI 0.25 to 0.33; *V*_Twin 2_(261) = 23,159, *P* < 0.001, *d* = 0.49, 95% CI 0.25 to 0.33), face preference (mean was significantly above chance level, highlighted as a dashed vertical line, *t*_Twin 1_(273) = 28.29, *P* < 0.001, *d* = 1.71, 95% CI 0.42 to 0.46; *t*_Twin 2_(261) = 26.31, *P* < 0.001, *d* = 1.63, 95% CI 0.41 to 0.45), and efficiency of visual exploration (number of objects explored during the first 10 s of trial).

Sample descriptive statistics are presented in Tables 1 and 2. As expected, we found a face bias significantly above chance level, for both face orienting (proportion of trials with first look at the face; one-sample two-tailed *V*_Twin 1_(273) = 25,558, *P* < 0.001, *d* = 0.54, 95% CI 0.25 to 0.33; *V*_Twin 2_(261) = 23,159, *P* < 0.001, *d* = 0.49, 95% CI 0.25 to 0.33) and preference (proportion of time spent looking at the face; *t*_Twin 1_(273) = 28.29, *P* < 0.001, *d* = 1.71, 95% CI 0.42 to 0.46; *t*_Twin 2_(261) = 26.31, *P* < 0.001, *d* = 1.63, 95% CI 0.41 to 0.45; Fig. 1). The univariate twin correlations for the three measures are presented in Table 3. Twin modelling assumptions (of equality of mean and variances across twin order and zygosity) were met for the three measures (Supplementary Tables 1, 3 and 5). For the efficiency of visual exploration (social and non-social objects), a univariate twin modelling analysis indicated no genetic influences (reported in Supplementary Table 2; for general information about the twin model fitting approach, also see Methods). Therefore, polygenic scores analysis involving efficiency of visual exploration were not conducted. Further, to simplify the multivariate twin analysis, we chose to only include the two phenotypes with genetic effects (univariate twin modelling analysis reported in Supplementary Tables 4 and 6).

**Table 1:** Descriptive statistics of the primary face looking measures. Statistics presented as mean (s.d.)/min–max

	Overall	MZ females	MZ males	DZ females	DZ males	Skewness
***N***	536	135	158	116	127	
**Age (in days)**	168 (9) 145–203	168 (9) 153–194	167 (8) 150–187	167 (8) 153–189	168 (10) 145–203	0.62
**No. valid trials**	5.77 (0.52) 4–7	5.76 (0.52) 4–7	5.75 (0.55) 4–6	5.75 (0.56) 4–6	5.82 (0.44) 4–6	−2.12
**Proportion missing gaze samples**	0.28 (0.13) 0–0.61	0.28 (0.13) 0.01–0.61	0.28 (0.12) 0.05–0.58	0.29 (0.13) 0.02–0.60	0.27 (0.14) 0–0.58	0.13
**Face orienting** (Proportion first look at the face)^*^	0.30 (0.19) 0–1	0.29 (0.19) 0–0.80	0.27 (0.2) 0–0.83	0.34 (0.19) 0–0.83	0.30 (0.19) 0–1	0.51
**Face preference** (Proportion on face)^*^	0.44 (0.14) 0.09–0.81	0.45 (0.14) 0.11–0.78	0.42 (0.15) 0.09–0.80	0.45 (0.13) 0.11–0.70	0.43 (0.14) 0.15–0.81	0.14
**Efficiency of visual exploration** **(No. objects explored, maximum** **5 during 10** **s)**	3.64 (0.54) 2–4.83	3.61 (0.54) 2.17–4.83	3.66 (0.52) 2.17–4.83	3.60 (0.56) 2–4.83	3.69 (0.55) 2.25–4.67	−0.34

^*^Chance level of face looking was 0.20 (1 in 5 objects was a face). MZ, monozygotic; DZ, dizygotic.

**Table 2:** Descriptive statistics of parent-reported development measures at 14 and 24 months. Statistics presented as mean (s.d.)/min–max

	*N* (*n* girls)	Age (in days)	Score (both sexes)	Females’ score	Males’ score	Skewness
**ECBQ self-regulation**						
**At 14** **months**	436 (196)	442 (20) 386–525	4.32 (0.58) 2.40–6.15	4.30 (0.59) 2.40–6.15	4.33 (0.56) 2.40–5.76	0.10
**At 24** **months**	358 (180)	755 (26) 707–920	4.56 (0.65) 2.73–6.25	4.69 (0.61) 2.73–6.00	4.44 (0.66) 2.80–6.25	−0.17
**ITC social communication**						
**At 14** **months**	418 (196)	444 (20) 387–525	34.93 (6.85) 11–51	35.84 (6.09) 20–51	34.13 (7.38) 11–51	−0.40
**CDI vocabulary**						
**At 14** **months**	420 (197)	443 (20) 386–502	82.52 (64.47) 1–332	92.90 (61.85) 1–305	73.35 (65.48) 3–332	1.16
**At 24** **months**	335 (168)	756 (23) 707–920	207.62 (156.91) 1–689	251.66 (143.71) 18–627	163.32 (157.55) 1–689	0.72

ECBQ, Early Childhood Behavior Questionnaire; ITC, Infant Toddler Checklist; CDI, Communicative Development Inventory.

**Table 3:** Twin correlation coefficients (95% CIs are shown in brackets) for the primary face looking measures, separate for MZ and DZ pairs

	*N* (twin pairs^*^)	Face orienting (proportion first look at the face)	Preference for face (proportion on face)	No. of objects explored (in 0–10 s)
**MZ**	155	0.20 [0.03 to 0.34]	0.46 [0.32 to 0.57]	0.05 [−0.12 to 0.22]
**DZ**	130	0.05 [−0.13 to 0.23]	0.21 [0.01 to 0.38]	0.15 [−0.03 to 0.31]

^*^Incomplete twin pairs

Correlations were derived from the univariate twin models where means and variances were equated across twin order and zygosity, and age and sex were included as covariates.

**Fig. 2:**
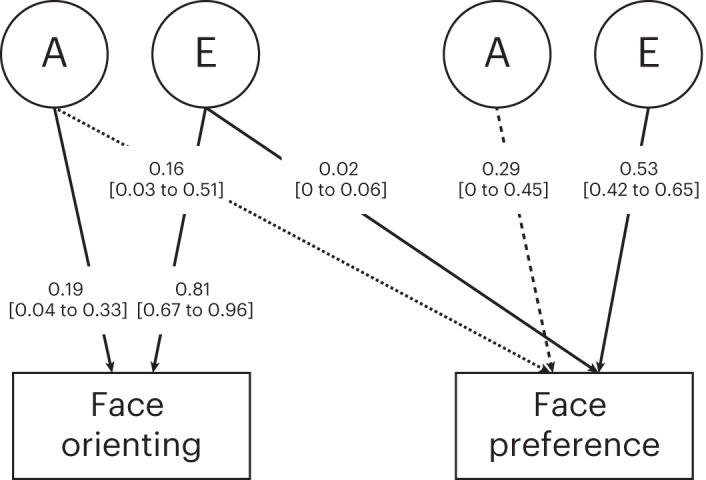
Schematic AE bivariate twin model for face orienting and face preference. Twin structural equation model-fitting was used to decompose the variance in face orienting and face preference into genetic (A) and unique environment (E) influences. Point estimates are shown with 95% CIs in brackets. The dotted line represents the shared genetic influences (significant), whereas the dashed line represents the unique genetic influences (non-significant).

Twin modelling assumptions for the bivariate twin analysis were met (equality of phenotypic and CTCT correlations across twin order and zygosity; Supplementary Table 7). The phenotypic correlation between face orienting and face preference was positive and moderate (*r*_Ph_ = 0.30, 95% CI 0.22 to 0.37, Δ*χ*^2^(Δd.f. 1) of 46.58, *P* < 0.001). A Cholesky bivariate twin model was used to examine genetic influences on face preference that were either unique to face preference or shared with face orienting (age and sex included as covariates). The AE model, that is, the model with additive genetic influences (A) and non-shared environment (E) and without shared environment influences (C), was selected and reported in Table 4 and Fig. 2 on the basis that it was the non-significant model (that is, did not have a significantly poorer fit compared with the ACE model, that is, the model with A, C, and E influences) with the lowest Akaike information criterion (AIC) value (for completeness, full ACE estimates are reported in Supplementary Table 8). The heritability of face orienting was 0.19 (95% CI 0.04 to 0.33), and the heritability of face preference was 0.46 (95% CI 0.33 to 0.57). The bivariate results showed that 97% of total E influencing face preference was unique to that variable and not shared with face orienting. Of the total genetic influences on face preference (A = 0.46, as above), 0.16 (95% CI 0.03 to 0.51) were shared with, and 0.29 (95% CI 0 to 0.45) were unique from, orienting to faces (Fig. 2). A follow-up analysis testing two nested models constraining the shared or the unique influences confirmed there was evidence for significant shared genetic influences between the two phenotypes and no evidence for significant unique genetic variance on face preference (Table 4).

**Table 4:** Bivariate twin model fit statistics for face orienting and face preference

Model	No. of parameters	−2LL	d.f.	AIC	Comparison model	Δ*χ*^2^	Δd.f.	*P* value
Fully sat.	32	1,151.37	1,040	−928.63	NA	NA	NA	NA
**ACE**								
ACE	15	1,167.82	1,057	−946.18	Fully sat.	16.45	17	0.492
**ACE-nested models**								
**AE**	**12**	**1,169.33**	**1,060**	−**950.67**	**ACE**	**1.51**	**3**	**0.680**
CE	12	1,175.34	1,060	−944.66	ACE	7.52	3	0.057
E	9	1,211.82	1,063	−914.18	ACE	44.00	6	<0.001
**AE-nested models**								
Unique path of 0	11	1,172.28	1,061	−949.72	AE	2.95	1	0.086
Shared path of 0	11	1,179.41	1,061	−942.59	AE	10.08	1	0.001

The best-fitting model was selected on the basis of non-significance (meaning that there was no decrement in fit compared with the saturated or the genetic model, indexed by the *χ*^2^ distribution) and the AIC fit statistic (which incorporates information about both explained variance and parsimoniousness).

The fully sat. model is the fully saturated model of the observed data, which models the means and variances for both variables, and the phenotypic and CTCT correlations between the two variables, separately for each twin in a pair and across zygosity.

In bold: the best-fitting model was non-significant with the lowest AIC.

−2LL, fit statistic, which is minus two times the log-likelihood of the data.

d.f., degrees of freedom.

AIC, fit statistic—lower values denote better model fits.

Δ*χ*^2^, difference in −2LL statistic between two models, distributed *χ*^2^.

Δd.f.*,* difference in degrees of freedom between two models.

### Association between face and eye-versus-mouth preferences

Given that infants’ preference for eyes (rather than mouth) when looking at faces has been found to correlate with face preference^24^ and has a substantial heritability (*h*^2^ = 0.57)^9^, we investigated the link between face preference (this study) and the previously analysed social looking phenotype. While there was a small but significant positive phenotypic association (*r*_Ph_ = 0.11, 95% CI 0.01 to 0.20, Δ*χ*^2^(Δd.f. 1) = 5.05, *P* = 0.025) between these phenotypes, there was no evidence for a genetic correlation (*r*_A_ = 0.10, 95% CI −0.13 to 0.31), and independent genetic factors contributed to eye (versus mouth) preference and face (versus object) preference (for full results, see Supplementary Result 1; this post hoc analysis was not pre-registered).

### Associations with polygenic scores

We found no evidence for associations between the face looking measures (face orienting and face preference) and polygenic scores for autism, ADHD, schizophrenia, depression and bipolar disorder (Supplementary Table 9).

### Longitudinal phenotypic associations

We found no evidence for associations between the looking measures and subsequent parent-reported measures of language, socio-communication and self-regulation (Table 5), with the exception of a statistically significant positive association between preference for the face at 5 months and receptive vocabulary (comprehension in the Communicative Development Inventory (CDI)) at 14 months (*β* = 0.14; 95% CI 0.03 to 0.25, *P* = 0.014; *R*^2^ = 0.018, *N* = 420).

**Table 5:** Results of multiple GEEs analyses with 14 months and 24 months parent-reported measures as outcome variables, the age, sex, and looking measures measured at 5 months as predictors, and twin pair identification as cluster-defining variable

ECBQ self-regulation at 14 months	*β*	s.e.	95% CI	Uncorrected *P* value	FDR threshold
Age (in days, scaled)	−0.08	0.06	−0.19 to 0.03	0.159	0.01
Sex (reference level: female)	0.06	0.12	−0.17 to 0.29	0.608	0.04
Face orienting (proportion of first look at the face)	0.07	0.05	−0.04 to 0.17	0.215	0.02
Preference for face (proportion on face)	<0.01	0.06	−0.11 to 0.11	0.935	0.05
No. of objects explored (in 0–10 s)	0.04	0.05	−0.05 to 0.14	0.377	0.03

ECBQ self-regulation at 24 months	*β*	s.e.	95% CI	Uncorrected *P* value	FDR threshold
Age (in days, scaled)	0.05	0.07	−0.09 to 0.19	0.475	0.03
**Sex (reference level: female)**	−**0.38**	**0.13**	−**0.63 to** −**0.13**	**0.003**	**0.01**
Face orienting (proportion of first look at the face)	0.06	0.06	−0.04 to 0.17	0.248	0.02
Face preference (proportion on face)	0.04	0.06	−0.08 to 0.15	0.548	0.05
No. of objects explored (in 0–10 s)	0.03	0.05	−0.07 to 0.14	0.530	0.04

ITC social communication at 14 months	*β*	s.e.	95% CI	Uncorrected *P* value	FDR threshold
Age (in days, scaled)	0.07	0.07	−0.07 to 0.21	0.348	0.04
Sex (reference level: female)	−0.27	0.12	−0.51 to −0.03	0.030	0.02
Face orienting (proportion of first look at the face)	−0.01	0.06	−0.12 to 0.11	0.918	0.05
Face preference (proportion on face)	0.13	0.06	0.02 to 0.24	0.026	0.01
No. of objects explored (in 0–10 s)	0.10	0.05	0.01 to 0.20	0.031	0.03

CDI receptive vocabulary at 14 months	*β*	s.e.	95% CI	Uncorrected *P* value	FDR threshold
**Age (in days, scaled)**	**0.15**	**0.06**	**0.04 to 0.27**	**0.007**	**0.01**
**Sex (reference level: female)**	**−0.34**	**0.13**	−**0.59 to** −**0.09**	**0.008**	**0.02**
Face orienting (proportion of first look at the face)	−0.01	0.05	−0.11 to 0.08	0.809	0.05
**Face preference (proportion on face)**	**0.14**	**0.06**	**0.03 to 0.25**	**0.014**	**0.03**
No. of objects explored (in 0–10 s)	0.07	0.05	−0.02 to 0.16	0.142	0.04

CDI expressive vocabulary at 24 months	*β*	s.e.	95% CI	Uncorrected *P* value	FDR threshold
**Age (in days, scaled)**	**0.21**	**0.05**	**0.11 to 0.31**	**<0.001**	**0.02**
**Sex (reference level: female)**	−**0.58**	**0.14**	−**0.85 to** −**0.32**	**<0.001**	**0.01**
Face orienting (proportion of first look at the face)	−0.01	0.05	−0.11 to 0.09	0.872	0.05
Face preference (proportion on face)	0.04	0.06	−0.08 to 0.15	0.550	0.04
No. of objects explored (in 0–10 s)	0.10	0.05	<0.01 to 0.20	0.046	0.03

For each model, all predictors were entered together; hence statistics represent unique contributions for each predictor. Adjustments were made for multiple comparisons using the FDR step-up approach. s.e., standard error.

In bold: significant predictors (*P* threshold in the FDR threshold column).

## Discussion

Attention preferences for social information, and looking at faces more specifically, have been suggested to play a key role in the development of social cognition^25^. Against this background, it is striking that we found substantial variability in infants’ attention to faces at an early point in life—before brain systems supporting social communication are fully developed (Fig. 1). As predicted, both face orienting at the beginning of the task and sustained face preference were heritable phenotypes. In contrast, we found no evidence for shared environment or biological sex effects in infants’ tendency to preferentially attend to faces versus non-social objects. The pattern of these results supports the view that, already during infancy, there is genetic variability to curate one’s visual environment via looking behaviours^9,10,20^, and that this applies to such broad categories as social versus non-social stimuli. This can be seen as a type of gene–environment correlation^26^ appearing very early in life. Active exposure to different visual environments entails different learning opportunities (active gene–environment correlation) and, because our point of gaze is visible to others, may evoke different reactions from other people (evocative gene–environment correlation). Visual environment selection by means of selective attention is one of the first ways infants can actively create or constrain their own visual experience and social interactions, emerging before other exploratory behaviours such as pointing, grasping or crawling to targets or partners.

Face preference, indexed by the looking time to the face relative to looking time to all objects in the scene, had a heritability of 46%, while face orienting, indexed by the proportion of first looks at the face, had a heritability of only 19%. A similar result was obtained when we used alternative related measures (that is, latency to look at the face; Supplementary Method 2). Given the adaptive and survival value of face orienting, it is not surprising that there is limited genetic variation linked to this phenotype. The small genetic variation associated with this phenotype might be related to face-selective processes as well as differences in general attention abilities^1^.

For efficiency of visual exploration, indexed by the number of objects (including face and non-face ones) an infant looked at during the first 10 s of the trial, we did not find evidence for familial effects (genetic or shared environment), and variability was best explained solely by unique environmental factors (which include measurement error). Perhaps our participants were too young to be displaying a stable measure of exploration, as indeed exploratory gaze patterns have been shown to be less consistent in infancy^27^, and/or our study was underpowered to detect subtle familial influences in this case (for details on power analysis in the study, see Supplementary Method 6).

Face orienting and face preference were moderately correlated, and there was evidence for shared genetic influences on face preference from face orienting (Fig. 2). While initial orienting and sustained attention to faces are hypothesized to be dissociated in terms of underlying brain networks (subcortical versus cortical networks^15^), it is possible that the observed shared variance is driven by subcortical processes influencing both phenotypes at this age^28^, by early emerging face-specific cortical structures influencing both face preference and orienting^29^, or by an inflation of the co-variance due to potential dependency of the measures (where you look first will probably influence to some extent your preferences at longer timescales).

Relatedly, we found that face preference was phenotypically and aetiologically largely independent from another heritable social looking phenotype in infancy: eye-versus-mouth looking^9^. This dissociation shows that social looking is not a unitary phenomenon, but is composed of multiple phenotypically and aetiologically distinct subdimensions^1,10^. Additionally, sensitivity analyses focusing on orienting to and preference for the second most looked at object (car) suggested no genetic effects for ‘car looking’ (Supplementary Method 4), supporting the idea that the genetic effects observed in the main analyses may be specific to the social/non-social contrast.

We predicted that both face preference and efficiency of visual exploration would be associated to later development (language/social communication and self-regulation, respectively). However, only the association between face preference and receptive vocabulary at 14 months was significant when applying stringent statistical criteria (Table 5, although it was not significant when controlled for gestational age instead of chronological age; Supplementary Method 8). We did not find an association between face preference and expressive vocabulary at 24 months. This could reflect the differences between the two scales (for further information, see Methods), but also equifinal developmental pathways^30^ where a temporary disadvantage in language development in some children (those looking less at faces at 5 months) disappears over time.

We did not find any associations between looking measures and genome-wide polygenic scores for autism or ADHD. While this might be reflecting true null effects, it is possible that the current polygenic scores do not yet have enough predictive power to detect these links.

The study has some notable limitations. First, the number of face pop-out trials in our study was six and increasing the number of trials could potentially lead to more stable measures of infant face orienting and objects exploration (though probably at a cost of increased participant attrition). Second, while the BATSS study included almost 30% of the same-sex twin population in the area, it reflected families with a higher socioeconomic status (SES) compared with the Stockholm normative population^23^. This needs to be considered in the generalizability of our results as genetic and environmental estimates may vary in samples where SES has a wider distribution^31^. Third, in contrast to the objective assessment of gaze behaviours at 5 months, later development was only assessed via questionnaires to parents. Finally, the current study used static images, and generalizability to dynamic or real-life stimuli is not known^1,32^.

In conclusion, our findings inform us about the aetiological influences on several important looking behaviours emerging early in infancy, and their developmental associations in the first 2 years of life. The results suggest two forms of gene–environment interplay unfolding at a micro-level in infancy. Firstly, because selective attention influences the input received, heritable preferences in infancy can be seen as a selection of the environment rooted in the individual’s biology. Further, looking at faces may cause reactions from the social partner. Both will have cascading effects in cognitive and social development.

## Methods

Three hundred and eleven families of same-sex twins were recruited to the BATSS^23^ and participated in an initial in-person assessment at 5 months old at Karolinska Institutet (data collection from April 2016 to February 2020) and participated in multiple follow-up online questionnaires at 14 months and 24 months (and 36 months, ongoing data collection at the time of submission of this work). BATSS was approved by the Regional Ethical Review Board in Stockholm and was conducted in accordance with all relevant ethical regulations and the Declaration of Helsinki. Parents gave informed consent to take part at each time point. A gift voucher of approximately €80 was given to each family at the initial in-person assessment. The main project sample description and inclusion criteria are described elsewhere^23^. Zygosity was estimated on the basis of DNA sampled from all infants. The current report uses a similar sample and the same twin statistical tools as a previous sample from our group^33^.

We excluded 28 twins from the total BATSS sample due to twin-to-twin transfusion syndrome, seizures at the time of birth, very low birth weight (<1.5 kg) or spina bifida^23^ (all parent reported). Furthermore, we excluded 23 infants because they did not complete the eye-tracking battery (technical reasons, time constraints, bad calibration or tiredness). Of the infants that completed the session, 35 infants did not provide enough valid trials and were excluded (valid trial criteria below). The final sample consisted of 536 infants (285 pairs with at least one individual with valid data; 251 pairs with valid data from both). The excluded (on the basis of invalid trials or not completing the eye-tracking assessment) and included infants did not statistically differ in terms of either parental education level, family income, sex or age.

### Eye-tracking protocol

To record infants’ gaze a Tobii TX300 eye-tracker was used (sampling rate of 120 Hz) with MATLAB (version R2013b, MathWorks) and Psychtoolbox (for stimuli presentation, version 3.0.12) with custom algorithms written for the Eurosibs study^34^. The task battery started with an initial five-point calibration and was followed by rotations of free-viewing of the face pop-out task (see below), dynamic scenes trials (mixture of social and abstract content^35^), gaze-contingent gap-overlap trials^36^, pupillary light reflex measurements^33^, and post-calibration sequences; the task battery lasted for about 10 min.

#### The face pop-out task

The face pop-out task^5,6,11,34,37^ was used to measure the various attentional processes involved in visual attention. It consisted of the presentation (fixed order, 20 s each) of a set of six different complex displays of objects, including a face (with direct eye gaze, three males and three females, counterbalancing ethnicity, and location of the face within the array) and four non-face competitors (including a ‘noise’ stimulus generated from the same face, a mobile phone, a bird and a car) (Supplementary Fig. 1). This type of display and the relatively long presentation time allow the study of the variation in active seeking of social information and attention flexibility by estimating the timing and preferential attention to faces and non-face stimuli.

Before each stimulus display, a small animation was presented and gaze-contingent methods started the presentation of the displays synchronous to the infant’s look at the central animation, ensuring the gaze was at the centre of the screen at the start of the trial. One infant viewed seven trials of the face pop-out (the first trial was repeated because the protocol had to be restarted)—all trials were included in the analysis.

### Computation of primary measures

Gaze data for each pop-out trial was processed using custom-written MATLAB scripts (analysis steps and areas of interest (AOIs), in Supplementary Method 1 and Supplementary Fig. 2). Any trials with a proportion of valid (non-missing) data less than 0.25 (25%), with the total duration of data for a trial less than 5 s, or where no look at an AOI was made, were excluded. Measures were averaged across trials for each infant if at least four valid trials were found. The distributions and boxplots for the proportion of valid trials where each AOI was the first AOI looked at and the proportion of looking time to each AOI (relative to all AOIs) can be seen in Supplementary Fig. 3.

Face orienting was operationalized as the proportion of first looks at the face (that is, the number of valid trials where the face was the first object looked at in relation to the number of valid trials), in deviation of the pre-registered plan of using a composite measure of the proportion of first looks at faces and the mean latency to look at a face. This decision was based on a modest correlation found between latency and proportion of first look at the face (standardized *β* = −0.33, *P* < 0.001) and unmet twin assumptions for the composite measure (driven by unmet equality of means and variances across zygosity and twin order for latency). However, sensitivity analyses with this composite led to a similar pattern of genetic univariate and bivariate findings (Supplementary Method 2).

Face preference was operationalized as the mean ratio of looking at the face, that is, the sum of looking time at the face AOI divided by the sum of looking time at all AOIs averaged across valid trials.

Efficiency of object exploration was operationalized as the mean number of objects looked at, averaged across valid trials. Each array of objects was presented for 20 s, a longer duration than in previous studies’ protocols^5,6,37^ where shorter versions were used (12 and 15 s). The longer duration meant that it was likely that infants looked at the five objects during the trial (in 45% of all trials the five objects were looked at). For this reason, and in deviation of the pre-registered plan, object exploration was estimated on the basis of only the first 10 s of the trial (in 22% of all trials, the five objects were looked at). The cut-off did not influence the results (Supplementary Method 3).

An analysis to contrast face orienting and preference (which reflect social versus non-social preferences) to the most attended (salient) non-social object (car) in the pop-out task, is reported in Supplementary Method 4.

#### Gaze quality measures

To control for potential effects of gaze quality in analyses, we estimated two gaze quality variables: the average proportion of missing data in the task (operationalized as the ratio of missing gaze per total data collected, averaged across valid trials) and the number of valid trials.

### Genome-wide polygenic scores

Genotyping of DNA samples was done using Infinium Global Screening Array (Illumina). Processing and quality control were done based on standard procedures and are described elsewhere^23^—for more details, see Supplementary Method 5. Polygenic scores were computed using the polygenic prediction via Bayesian regression and continuous shrinkage priors method^38^, based on the most recent and largest (at the time of calculation of the scores, November 2020–March 2021) genome-wide association studies for ADHD^39^, autism^40^, bipolar disorder^41^, major depressive disorder^42^ and schizophrenia^43^. For this analysis, the first ten principal components of ancestry were included as covariates.

### Parent-rated developmental questionnaires

#### Social communication

The Communication and Symbolic Behavior Scales Developmental Profile Infant Toddler Checklist (ITC^44^) was used to measure socio-communicative behaviours (as indexed by the total raw score) at 14 months. A lower score is indicative of communication difficulties.

#### Language

The Swedish Early Communicative Development Inventory (CDI^45,46^), adapted from the Macarthur-Bates Communicative Development Inventory, was used to measure vocabulary at 14 months (the words and gestures form) and 24 months (the words and sentences form). In line with our study of eye-versus-mouth looking with the same sample^9^, we used receptive vocabulary (number of words the child understands) at 14 months, and we used expressive vocabulary (number of words the child understands and says) at 24 months. At 14 months, the production scale produces substantial floor effects. At 24 months, the CDI reliably measures individual differences in language production and infants’ receptive vocabulary is typically too large to be quantified by parents, and is not included in the words and sentences form.

#### Self-regulation

The Early Childhood Behavior Questionnaire (ECBQ^47^) was used to measure self-regulation (as indexed by the effortful control scale of the questionnaires) at 14 months (short-form, 107 items) and 24 months (very-short-form, 36 items).

### Analysis plans

An analysis plan for this study was registered in Open Science Framework^19^ on 20 August 2021 (before data cleaning and analysis). R software (version 4.0.0) was used for all data computation and analyses. A power analysis was conducted before the data collection (Supplementary Method 6). All statistical testing were two-sided.

To test face orienting and preference against chance level, one-sample two-tailed tests were conducted for twins independently (Wilcoxon signed rank test was used for face orienting and *t*-test for face preference; because only the latter followed a normal distribution tested with the Shapiro–Wilk normality test). Effect sizes were estimated on the basis of mean minus the chance level (0.2) divided by the standard deviation (s.d.).

For twin models, the OpenMx package^48^ (version 2.18.1 with NPSOL optimizer) with full-information maximum likelihood estimation was used, which allows for partially complete pairs (one twin missing) to be included.

For each looking measure, both saturated models (which test for the assumptions of equality of mean and variances across twin order and zygosity) and univariate twin models were fitted separately and reported in Supplementary Tables 1–6. A bivariate saturated model (which tested the assumption of equal phenotypic and CTCT correlations by zygosity and between twins) and a bivariate twin model (Cholesky decomposition) were fitted on the two variables, namely face orienting and face preference. As noted in the main text, twin structural equation model-fitting is a statistical approach involving the decomposition of variance in a phenotype/set of phenotypes into genetic (A), shared environment (C) and unique environment (E) influences. A full model (including A, C and E) was evaluated against several possible nested (simpler) models^49^. The best-fitting nested model was defined as the non-significant model with the lowest AIC value. When a nested model is significant, it means that it has poorer fit than the full model, indexed by the *χ*^2^ distribution, and hence should be excluded (this entails that the selected nested model is always statistically non-significant^50^). The AIC fit statistic incorporates information about both explained variance and parsimoniousness; the lowest value corresponds to the best model. Twin and CTCT correlations were derived from the constrained saturated models, in which means, variances, phenotypic and CTCT correlations were constrained to be equal across twin order and zygosity. When the pattern of correlations suggested non-additive genetic effects (D; MZ correlation more than twice the DZ correlation), a decision was made to report an ACE model (the model including A, C, and E) rather than an ADE model (the model including A, D, and E) to our data due to sample size (for the ADE bivariate model results, see Supplementary Table 8). In accordance with the standard reporting for twin research^51,52^, we report CIs for each component included in the best-fitting model, whether the CI overlaps with zero shows whether the component is statistically significant.

Association analyses were conducted, whenever possible, with the whole twin sample (that is, including both twins in a pair, including pairs with one twin missing) using linear regression models implemented as generalized estimating equations (GEEs; using the drgee package^53^), with cluster-robust standard errors to account for family relatedness, to derive *β* estimates and *P* values. All measures were scaled so that *β* estimates were standardized. Effect sizes (Δ*R*^2^) were calculated on the basis of comparing the *R*^2^ of the null model (that is, the model with only covariates included) and of corresponding models. When controlling for multiple testing (when testing the longitudinal phenotypic associations) a false discovery rate (FDR) step-up approach was used across analyses using the same outcome.

Chronological age (in days) and sex were always included in twin models and added to the GEE models as covariates. Associations between the gaze quality covariates (proportion missing gaze and number of valid trials) and the gaze-based primary visual attention measures were tested within the GEE framework (one linear model with both covariates as predictors were run for each primary variable). If statistically significant, the gaze quality measures were regressed out from the main dependent variables before all other analyses. Eye-tracking accuracy and precision were also tested as additional gaze quality covariates in a sensitivity analysis presented in Supplementary Method 7. Corrected age (age estimated on the basis of birth date and gestational age at birth) was included in twin models and added to the GEE models as covariates (in replacement of chronological age) in a sensitivity analysis reported in Supplementary Method 8.

### Reporting summary

Further information on research design is available in the Nature Portfolio Reporting Summary linked to this article.

### Supplementary information

Supplementary Information Supplementary Tables 1–36, Methods 1–7, Result 1 and Figs. 1–3.

Reporting Summary

Peer Review File

## Data Availability

Unrestricted sharing of pseudonymized personal data was not specified in the study ethics application; hence, data are not uploaded to a public repository. However, data are available from T.F.Y. (terje.falck-ytter@psyk.uu.se) on reasonable request. Request will be responded to within 1 week. Sharing pseudonymized (coded) data from the study will require a data sharing agreement according to Swedish and EU law.

## Code availability

The face pop-out pre-processing workflow (implemented in MATLAB and described in Supplementary Method 1) is part of a shared agreement and available from L.M. on reasonable request. The statistical analysis scripts (implemented in R) are publicly available in OSF (https://osf.io/zseh2/). The R code for the raincloud plot visualizations (Fig. 1 and Supplementary Fig. 3) has been adapted from Allen et al.^54^.
