# Supplementary material for: Infants’ looking preferences for social versus non-social objects reflect genetic variation
Source: Nat Hum Behav. 2023 Nov 27;8(1):115–24. doi: 10.1038/s41562-023-01764-w (PMC10810753; doi:10.1038/s41562-023-01764-w)
Supplement: Supplementary file 1 — Supplementary Tables 1–36, Methods 1–7, Result 1 and Figs. 1–3. [file 41562_2023_1764_MOESM1_ESM.pdf]

nature human behaviour

Article

https://doi.org/10.1038/s41562-023-01764-w

Infants' looking preferences for social versus non-social objects reflect genetic variation

In the format provided by the authors and unedited

Supplementary Information

Supplementary Tables ... 2
Supplementary Figures ... 11
Supplementary Methods 1. Steps in gaze offline pre-processing: ... 12
Supplementary Methods 2. Sensitivity analyses for face orienting ... 15
Supplementary Methods 3. Sensitivity analyses for number of objects explored (in 0-20 seconds) ... 18
Supplementary Methods 4. Analyses for non-social attention ... 21
Supplementary Methods 5. Methods for the computation of genome-wide polygenic scores ... 26
Supplementary Methods 6. Power analysis ... 27
Supplementary Methods 7. Sensitivity Analyses for additional gaze quality metrics ... 27
Supplementary Methods 8. Sensitivity Analyses for corrected age ... 34
Supplementary Results 1. Analyses for the association between preference for faces and preferential looking within faces (eye versus mouth).. 41

Supplementary Tables

Supplementary Table 1. Univariate saturated model for the number of objects explored (in 0-10 seconds) including covariates (age and sex). The χ2 distribution and associated p-value were used to test the effect of the covariates (there was evidence of an effect if there was a significant decrement in fit compared to the saturated model) and twin model-fitting assumptions (assumptions were violated if there was a significant decrement in fit compared to the saturated model).

					**Comparative fit with saturated model**		
**Model**	**# Parameters**	**-2LL**	***df***	**AIC**	**Δ χ2**	**Δ *df***	**P value**
Saturated	12	1507.21	524	459.21	-	-	-
Age	11	1507.21	525	457.21	0.01	1	.941
Sex	11	1509.15	525	459.15	1.94	1	.163
1.	10	1512.52	526	460.52	5.32	2	.070
2.	9	1512.53	527	458.53	5.32	3	.150
3.	7	1514.50	529	456.50	7.30	5	.200
4.	6	1514.93	530	454.93	7.72	6	.259

Model definitions. The baseline model is the fully saturated model of the observed data, which models the means and variances separately for each twin in a pair and across zygosity. **Age.** Testing the significance of age, **Sex.** Testing the significance of sex, **1.** Equating means across twins within a pair, **2.** Equating means across zygosity, **3.** Equating variances across twins within a pair, and **4.** Equating variances across zygosity (i.e., the constrained saturated model).
-2LL = fit statistic, which is minus two times the log-likelihood of the data.
df = degrees of freedom
AIC, fit statistic. Lower values denote better model fits.
Δ χ2 = difference in −2LL statistic between two models, distributed χ2.
Δ *df* = difference in degrees of freedom between two models.

Supplementary Table 2. Univariate twin model fit statistics and parameter estimates for number of objects explored (in 0-10 seconds). The best fitting model was selected based on non-significance (meaning that there was no decrement in fit compared to the saturated or the genetic

model, indexed by the χ2 distribution) and the AIC fit statistic (Akaike information criterion, which incorporates information about both explained variance and parsimoniousness).

Model	# parameters	-2LL	*df*	AIC	Comparison model	Δ χ2	Δ *df*	P value	A	C	E
**Fully Sat**	12	1507.21	524	459.21	NA	-	-	-	-	-	-
**ACE**	6	1515.50	530	455.50	Fully Sat.	8.29	6	.218	0	.10	.90
**AE**	5	1516.41	531	454.41	ACE	0.91	1	.341	.10	-	.90
**CE**	**5**	**1515.50**	**531**	**453.50**	**ACE**	**0**	**1**	**> .999**	**-**	**.10 [0, .22]**	**.90 [.78, 1]**
**E**	4	1518.00	532	454.00	ACE	2.50	2	.286	-	-	1

In bold: the best-fitting model was the non-significant with the lowest AIC.
-2LL = fit statistic, which is minus two times the log-likelihood of the data.
df = degrees of freedom
AIC, fit statistic. Lower values denote better model fits.
Δ χ2 = difference in −2LL statistic between two models, distributed χ2.
Δ *df* = difference in degrees of freedom between two models.

Supplementary Table 3. Univariate saturated model for face orienting (proportion of first look to faces) including covariates (age and sex). The χ2 distribution and associated p-value were used to test the effect of the covariates (there was evidence of an effect if there was a significant decrement in fit compared to the saturated model) and twin model-fitting assumptions (assumptions were violated if there was a significant decrement in fit compared to the saturated model).

					Comparative fit with saturated model		
**Model**	**# Parameters**	**-2LL**	***df***	**AIC**	**Δ χ2**	**Δ *df***	**P value**
Saturated	12	-260.80	524	-1308.80	-	-	-
Age	11	-258.49	525	-1308.49	2.31	1	.129

Sex	11	-258.94	525	-1308.94	1.86	1	.173
1.	10	-260.14	526	-1312.14	0.66	2	.718
2.	9	-254.83	527	-1308.83	5.97	3	.113
3.	7	-252.42	529	-1310.42	8.38	5	.136
4.	6	-252.27	530	-1312.27	8.53	6	.202

Model definitions. The baseline model is the fully saturated model of the observed data, which models the means and variances separately for each twin in a pair and across zygosity. **Age.** Testing the significance of age, **Sex.** Testing the significance of sex, **1.** Equating means across twins within a pair, **2.** Equating means across zygosity, **3.** Equating variances across twins within a pair, and **4.** Equating variances across zygosity (i.e., the constrained saturated model).
-2LL = fit statistic, which is minus two times the log-likelihood of the data.
df = degrees of freedom
AIC, fit statistic. Lower values denote better model fits.
Δ χ2 = difference in −2LL statistic between two models, distributed χ2.
Δ *df* = difference in degrees of freedom between two models.

Supplementary Table 4. Univariate twin model fit statistics and parameter estimates for face orienting (proportion of first look to faces). The best fitting model was selected based on non-significance (meaning that there was no decrement in fit compared to the saturated or the genetic model, indexed by the χ2 distribution) and the AIC fit statistic (Akaike information criterion, which incorporates information about both explained variance and parsimoniousness).

**Model**	**# parameters**	**-2LL**	***df***	**AIC**	**Comparison model**	**Δ χ2**	**Δ *df***	**P value**	**A**	**C/D**	**E**
**Fully Sat**	12	-260.80	524	-1308.80	NA	-	-	-	-	-	-
**ACE**	6	-252.05	530	-1312.05	Fully Sat.	8.75	6	.188	.18	0	.82
**AE**	**5**	**-252.05**	**531**	**-1314.05**	**ACE**	**0.00**	**1**	**> .999**	**.18 [.03, .32]**	**-**	**.82 [.68, .97]**
**CE**	5	-250.87	531	-1312.87	ACE	1.18	1	.278	-	.13	.87
**E**	4	-246.42	532	-1310.42	ACE	5.63	2	.060	-	-	1
**ADE**	6	-252.27	530	-1312.27	Fully Sat.	8.53	6	.202	.01	.19	.81

**AE**	5	-252.05	531	-1314.05	ADE	0.22	1	.638	.18	-	.82
**E**	4	-246.42	532	-1310.42	ADE	5.85	2	.054	-	-	1

In bold: the best-fitting model was the non-significant with the lowest AIC.
-2LL = fit statistic, which is minus two times the log-likelihood of the data.
df = degrees of freedom
AIC, fit statistic. Lower values denote better model fits.
Δ χ2 = difference in −2LL statistic between two models, distributed χ2.
Δ *df* = difference in degrees of freedom between two models.

Supplementary Table 5. Univariate saturated model for the face preference (proportion looking time on face) including covariates (age and sex). The χ2 distribution and associated p-value were used to test the effect of the covariates (there was evidence of an effect if there was a significant decrement in fit compared to the saturated model) and twin model-fitting assumptions (assumptions were violated if there was a significant decrement in fit compared to the saturated model).

					**Comparative fit with saturated model**		
**Model**	**# Parameters**	**-2LL**	***df***	**AIC**	**Δ χ2**	**Δ *df***	**P value**
Saturated	12	1463.71	524	415.71	-	-	-
**Age**	**11**	**1470.19**	**525**	**420.19**	**6.48**	**1**	**.011**
Sex	11	1466.57	525	416.57	2.86	1	.091
1.	10	1466.16	526	414.16	2.45	2	.294
2.	9	1466.17	527	412.17	2.46	3	.483
3.	7	1467.85	529	409.85	4.14	5	.529
4.	6	1468.96	530	408.96	5.25	6	.512

Model definitions. The baseline model is the fully saturated model of the observed data, which models the means and variances separately for each twin in a pair and across zygosity. **Age.** Testing the significance of age, **Sex.** Testing the significance of sex, **1.** Equating means across twins within a pair, **2.** Equating means across zygosity, **3.** Equating variances across twins within a pair, and **4.** Equating variances across zygosity (i.e., the constrained saturated model).

In bold: models with a significant poorer fit compared with the saturated model.
-2LL = fit statistic, which is minus two times the log-likelihood of the data.
df = degrees of freedom
AIC, fit statistic. Lower values denote better model fits.
Δ χ2 = difference in −2LL statistic between two models, distributed χ2.
Δ *df* = difference in degrees of freedom between two models.

Supplementary Table 6. Univariate twin model fit statistics and parameter estimates for face preference (proportion looking time on face). The best fitting model was selected based on non-significance (meaning that there was no decrement in fit compared to the saturated or the genetic model, indexed by the χ2 distribution) and the AIC fit statistic (Akaike information criterion, which incorporates information about both explained variance and parsimoniousness).

Model	# parameters	-2LL	*df*	AIC	Comparison model	Δ χ2	Δ *df*	P value	A	C/D	E
**Fully Sat**	12	1463.71	524	415.71	NA	-	-	-	-	-	-
**ACE**	6	1469.00	530	409.00	Fully Sat.	5.29	6	.507	.45	0	.55
**AE**	**5**	**1469.00**	**531**	**407.00**	**ACE**	**0.00**	**1**	**> .999**	**.45 [.33, .56]**	**-**	**.55 [.44, .68]**
**CE**	5	1474.29	531	412.29	ACE	5.28	1	.022	-	.36	.64
**E**	4	1507.67	532	443.67	ACE	38.66	2	< .001	-	-	1
**ADE**	6	1468.96	530	408.96	Fully Sat.	5.25	6	.512	.37	.08	.54
**AE**	5	1469.00	531	407.00	ADE	0.05	1	.831	.45	-	.55
**E**	4	1507.67	532	443.67	ADE	38.71	2	< .001	-	-	1

In bold: the best-fitting model was the non-significant with the lowest AIC.
-2LL = fit statistic, which is minus two times the log-likelihood of the data.
df = degrees of freedom
AIC, fit statistic. Lower values denote better model fits.
Δ χ2 = difference in −2LL statistic between two models, distributed χ2.
Δ *df* = difference in degrees of freedom between two models.

Supplementary Table 7. Assumption testing for the bivariate model between face orienting (proportion of first look to faces) and face preference. The χ2 distribution and associated p-value was used to test the twin model-fitting assumptions (assumptions were violated if there was a significant decrement in fit compared to the saturated model).

					**Comparative fit with saturated model**		
**Model**	**# Parameters**	**-2LL**	***df***	**AIC**	**Δ χ2**	**Δ *df***	**P value**
Saturated	32	1151.37	1040	-928.63	-	-	-
5.	15	1165.79	1057	-948.21	14.42	17	.637

Model definitions. The Fully Sat. model is the fully saturated model of the observed data, which models the means and variances for both variables, and the phenotypic and cross-twin-cross-trait correlations between the two variables, separately for each twin in a pair and across zygosity. **5.** In the Bivariate model fitting, the constrained saturated model equates means, variances, phenotypic and cross-twin-cross-trait correlations across twins within a pair and across zygosity, for both variables of interest.
The best-fitting model (in bold) was the non-significant and most parsimonious model, as well as the one with the lowest AIC.
-2LL = fit statistic, which is minus two times the log-likelihood of the data.
df = degrees of freedom
AIC, fit statistic. Lower values denote better model fits.
Δ χ2 = difference in −2LL statistic between two models, distributed χ2.
Δ *df* = difference in degrees of freedom between two models.

Supplementary Table 8. ACE and ADE Bivariate twin model fit statistics and estimates between face orienting (proportion of first look to faces) and face preference. The best fitting model was selected based on non-significance (meaning that there was no decrement in fit compared to the saturated or the genetic model, indexed by the χ2 distribution) and the AIC fit statistic (Akaike information criterion, which incorporates information about both explained variance and parsimoniousness).

**Model**	**# Parameters**	**-2LL**	***df***	**AIC**	**Comparison model**	**Δ χ2**	**Δ *df***	**P value**	**A.1**	**A.12**	**A.2**	**C/D.1**	**C/D.12**	**C/D.2**	**E.1**	**E.12**	**E.2**
**Fully Sat**	32	1151.37	1040	-928.63	NA	NA	NA	NA	-	-	-	-	-	-	-	-	-

**ACE**	15	1167.82	1057	-946.18	Fully Sat.	16.45	17	.492	.15	.37	0	.04	.08	0	.81	.02	.53
**ADE**	15	1167.91	1057	-946.09	Fully Sat.	16.54	17	.486	< .01	.24	0	.21	.23	0	.79	.02	.52
**AE**	**12**	**1169.33**	**1060**	**-950.67**	**ADE**	**1.43**	**3**	**.700**	**.19 [0.04, 0.33]**	**.16 [0.03, 0.51]**	**.29 [0, 0.45]**	-	-	-	**.81 [0.67, 0.96]**	**.02 [0, 0.06]**	**.53 [0.42, 0.65]**
**E**	9	1211.82	1063	-914.18	ADE	43.92	6	< .001	-	-	-	-	-	-	1	.09	.91

Model definitions. The Fully Sat. model is the fully saturated model of the observed data, which models the means and variances for both variables, and the phenotypic and cross-twin-cross-trait correlations between the two variables, separately for each twin in a pair and across zygosity.
In bold: the best-fitting model was the non-significant with the lowest AIC.
-2LL = fit statistic, which is minus two times the log-likelihood of the data.
df = degrees of freedom
AIC, fit statistic. Lower values denote better model fits.
$\Delta \chi2$ = difference in −2LL statistic between two models, distributed $\chi2$.
Δ *df* = difference in degrees of freedom between two models.
A = additive genetic influences
C = shared environment influences
D = non-additive genetic influences
E = non-shared environment influences
A-C/D-E.1 = variance on Phenotype 1 (proportion of first look to face)
A-C/D-E.12 = variance on Phenotype 2 (face preference) that is shared with Phenotype 1 (proportion of first look to face)
A-C/D-E.2 = unique variance on Phenotype 2 (face preference)

Supplementary Table 9. Summary of the two Generalized Estimating Equations models including genome-wide polygenic scores (GPSs) for autism, ADHD, bipolar disorder, major depressive disorder, and schizophrenia, 10 principal component of ancestry, and age and sex, as

predictors of face orienting (proportion of first look to faces) or face preference, with twin pair id as cluster-defining variable. Significant predictors are in bold (adjustments were made for multiple comparisons setting the alpha threshold for the number of outcomes, = .025).

**Face orienting (Proportion first look to face)**	**Beta**	**95% CI**	**Uncorrected *p*-value**
Age (in days)	<0.01	[-0.00, 0.00]	0.295
Sex	-0.03	[-0.06, 0.01]	0.111
GPS autism	-0.01	[-0.03, 0.02]	0.636
GPS ADHD	-0.01	[-0.03, 0.01]	0.269
GPS bipolar disorder	0.00	[-0.02, 0.03]	0.771
GPS major depression disorder	0.02	[-0.00, 0.04]	0.064
GPS schizophrenia	-0.02	[-0.05, 0.00]	0.054
PC 2	0.10	[-3.10, 3.29]	0.953
PC 3	-1.73	[-3.40, -0.05]	0.043
PC 4	2.38	[-1.12, 5.87]	0.182
PC 5	3.20	[-0.68, 7.08]	0.106
PC 6	-0.31	[-3.90, 3.28]	0.865
PC 7	-0.84	[-4.04, 2.36]	0.607
PC 8	-0.56	[-3.69, 2.57]	0.726
PC 9	3.39	[-0.28, 7.05]	0.070
PC 10	0.43	[-2.72, 3.58]	0.789
**PC 11**	**4.83**	**[1.18, 8.48]**	**0.009**
**Face preference (Proportion on face)**	**Beta**	**95% CI**	**Uncorrected *p*-value**
**Age (in days)**	**0.01**	**[0.00, 0.02]**	**0.008**
Sex	-0.17	[-0.35, 0.02]	0.085
GPS autism	-0.03	[-0.14, 0.09]	0.645

GPS ADHD	0.00	[-0.11, 0.11]	0.980
GPS bipolar disorder	-0.04	[-0.20, 0.12]	0.608
GPS major depression disorder	0.04	[-0.07, 0.15]	0.483
GPS schizophrenia	0.06	[-0.08, 0.19]	0.396
PC 2	-14.45	[-29.06, 0.17]	0.053
PC 3	-0.57	[-11.48, 10.35]	0.919
PC 4	-23.23	[-46.63, 0.16]	0.052
PC 5	-12.59	[-34.32, 9.14]	0.256
PC 6	-5.70	[-24.94, 13.53]	0.561
PC 7	-10.52	[-30.47, 9.42]	0.301
PC 8	4.93	[-13.99, 23.86]	0.609
PC 9	20.12	[-0.37, 40.61]	0.054
PC 10	3.62	[-17.67, 24.90]	0.739
PC 11	17.41	[-1.77, 36.58]	0.075

Supplementary Figures

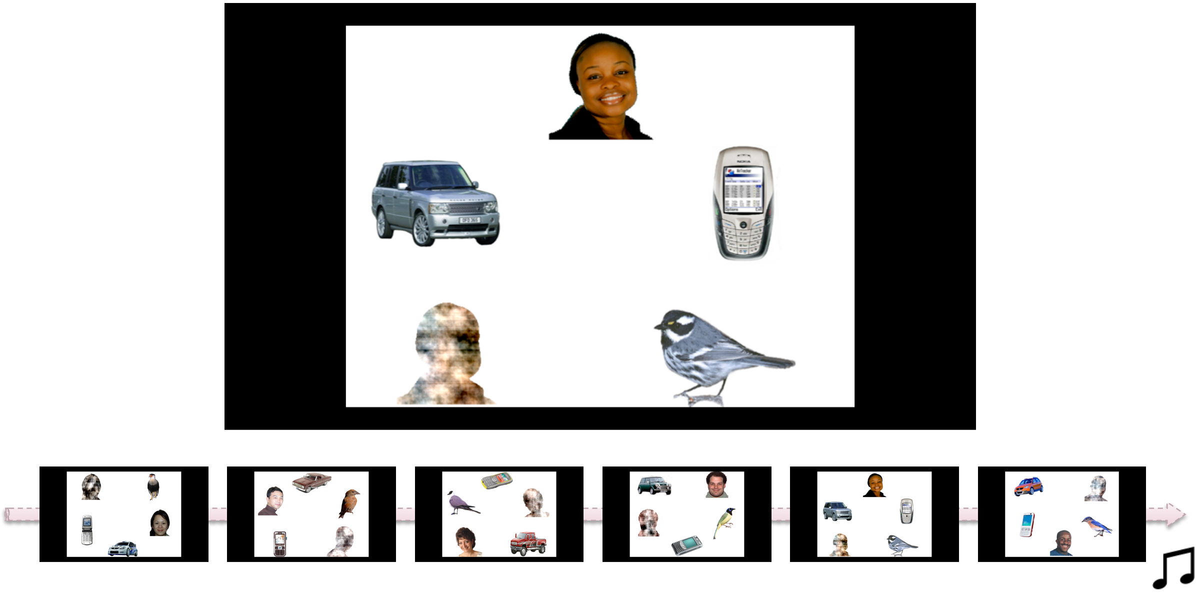

Supplementary Figure 1. Scheme of the face pop-out experimental stimuli. The face pop-out task included 6 trials presented for 20 seconds each in a fixed order. Each trial showed a complex display of objects including a face (with direct eye-gaze; 3 male, 3 female; counterbalancing ethnicity and location of the face within the array) and 4 non-face competitors (including a 'noise' stimulus generated from the same face, a mobile phone, a bird, and a car).

Supplementary Methods 1. Steps in gaze offline pre-processing:

1) Continuous raw eye tracking data was resampled to 60Hz
2) Off-screen gaze was marked as missing gaze
3) X and Y coordinates were averaged when binocular data was present (data from one eye was used when one eye was missing)
4) Large AOIs (centre, face, noise, car, bird, phone) were defined around each stimulus (see **Supplementary Figure 2**).
    a. Raw data was assigned to AOIs, by coding logical vectors (n=5) of gaze samples inside (1) and outside (0) each AOI
    b. AOI vectors were interpolated to fill in gaps of missing data shorter than 200ms (i.e. recode from 0 to 1)
    c. Any runs of samples in an AOI vector with a length less than 50ms were recoded to 0 (trigger tolerance for AOI activation), to ensure that a minimum of 50ms of gaze data was accumulated in an AOI for a look to be computed.
5) For each trial and AOI we computed:
    a. Whether the AOI was looked at, coded as true if at least a contiguous run of samples with 50 ms was identified inside the AOI.
    b. The latency of the first look (if a. was true), coded as the duration from the start of the trial to the first sample in the AOI.
    c. The looking ratio in the AOI, coded as the number of samples in AOI per number of samples in all AOIs.
6) For each trial we also computed:
    a. The number of objects looked at, coded as the count of AOIs looked at (i.e., 5. a. was true).

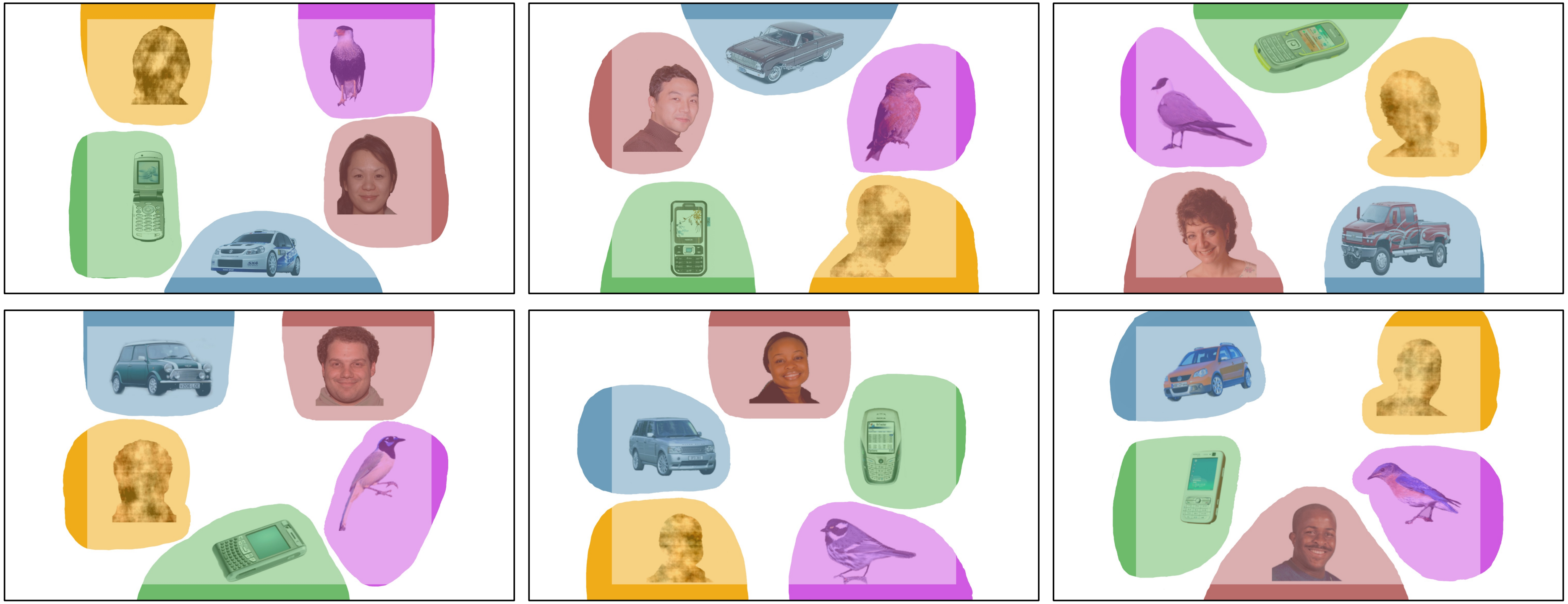

Supplementary Figure 2. Stimuli arrays of the face pop-out task with overlapping Areas of interest.

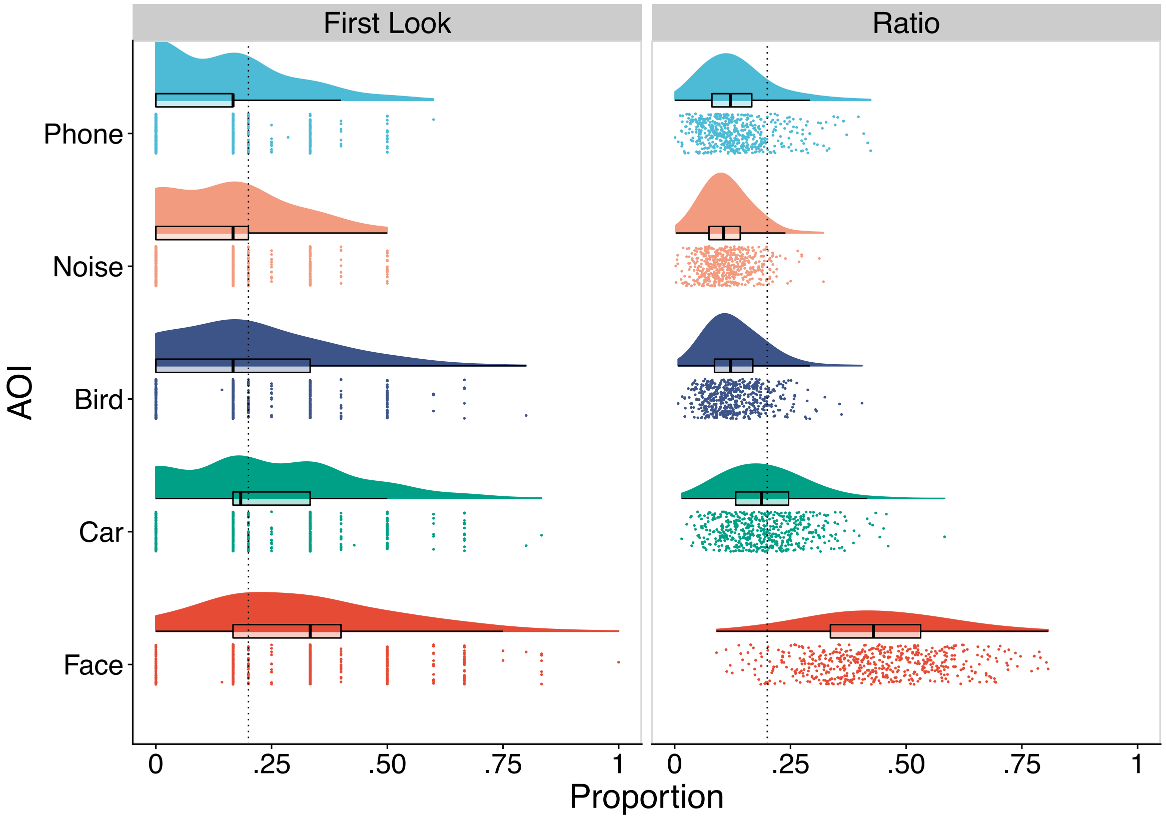

Supplementary Figure 3. Raincloud plots [1] *(center lines represent the median; box limits represent upper and lower quartile; whiskers represent 1.5x interquartile range; outliers are not presented) for 536 5-month-old infants' measures. Here we show, for each object, the proportion of trials of first looks towards that object (first look) and proportion of time looking at that object throughout the trial, relative to time looking at all objects (ratio). Chance levels highlighted as dashed vertical lines.*

[1] Allen, M., Poggiali, D., Whitaker, K., Marshall, T. R. & Kievit, R. A. Raincloud plots: a multi-platform tool for robust data visualization. *Wellcome Open Res* 4, 63 (2019).

Supplementary Methods 2. Sensitivity analyses for face orienting

To fulfil the pre-registered analyses plan, we report here the univariate analyses for face orienting operationalized as a composite average score between the proportion of trials that the infant looked at the face as the first AOI and the mean latency to look at the face (latencies shorter than 120 ms were excluded prior to averaging across valid trials) – see more details in the Methods section "Computation of primary measures"). The latency score was reversed and both measures were z-scored before averaging them. A higher score on this measure indicates a faster and larger face orienting. The twin correlations suggested genetic influences on the composite score (ICC_{MZ}=.25, 95% CI [.08, .39]; ICC_{DZ}=.13, 95% CI [-.06, .30]). The twin models confirmed these genetic effects but the assumptions for twin modelling were not met.

Supplementary Table 10. Descriptive statistics for face orienting composite score and latency to look at the face AOI.

	Overall	MZ Females	MZ Males	DZ Females	DZ Males	Skewness
**N (twins)**	536	135	158	116	127	
**Face orienting composite**	0 (0.81)<br>-2.50-2.41	-0.05 (0.78)<br>-2.14-1.88	-0.10 (0.83)<br>-2.18-2.07	0.17 (0.84)<br>-1.93-2.05	0.02 (0.79)<br>-2.50-2.41	0.05
**Latency (ms)**	2.93 (1.82)<br>0.41-10.80	3.00 (1.73)<br>0.56-8.13	3.06 (1.93)<br>0.41-10.21	2.71(1.77)<br>0.50-9.01	2.90 (1.82)<br>0.54-10.80	1.06

Supplementary Table 11. Saturated model for the composite score of face orienting (proportion of first look to face and latency), including covariates (age and sex). Note this is not the same measure that it was reported in the manuscript (only proportion of first look to face). The χ2 distribution and associated p-value were used to test the effect of the covariates (there was evidence of an effect if there was a significant decrement in fit compared to the saturated model) and twin model-fitting assumptions (assumptions were violated if there was a significant decrement in fit compared to the saturated model).

					**Comparative fit with saturated model**		
**Model**	**# Parameters**	**-2LL**	***df***	**AIC**	**Δ χ2**	**Δ *df***	**P value**
Saturated	12	1484.49	524	436.49	-	-	-
**Age**	**11**	**1494.07**	**525**	**444.07**	**9.59**	**1**	**.002**
Sex	11	1486.12	525	436.12	1.64	1	.201
1.	10	1489.12	526	437.12	4.63	2	.099
**2.**	**9**	**1493.34**	**527**	**439.34**	**8.86**	**3**	**.031**
**3.**	**7**	**1499.60**	**529**	**441.60**	**15.11**	**5**	**.010**
**4.**	**6**	**1499.61**	**530**	**439.61**	**15.13**	**6**	**.019**

Model definitions. The baseline model is the fully saturated model of the observed data, which models the means and variances separately for each twin in a pair and across zygosity. **Age.** Testing the significance of age, **Sex.** Testing the significance of sex, **1.** Equating means across twins within a pair, **2.** Equating means across zygosity, **3.** Equating variances across twins within a pair, and **4.** Equating variances across zygosity (i.e., the constrained saturated model).
In bold: models with a significant poorer fit compared with the saturated model.
-2LL = fit statistic, which is minus two times the log-likelihood of the data.
df = degrees of freedom
AIC, fit statistic. Lower values denote better model fits.
Δ χ2 = difference in −2LL statistic between two models, distributed χ2.
Δ *df* = difference in degrees of freedom between two models.

Supplementary Table 12. Univariate twin model fit statistics and parameter estimates for the composite score of face orienting. Note this is not the same measure that it was reported in the manuscript (proportion of first look to face). The best fitting model was selected based on non-significance (meaning that there was no decrement in fit compared to the saturated or the genetic model, indexed by the χ2 distribution) and the AIC fit statistic (Akaike information criterion, which incorporates information about both explained variance and parsimoniousness).

Model	# parameters	-2LL	*df*	AIC	Comparison model	Δ χ2	Δ *df*	P value	A	C	E
**Fully Sat**	12	1484.49	524	436.49	NA	-	-	-	-	-	-
**ACE**	6	1499.61	530	439.61	Fully Sat.	15.13	6	.019	.23	.02	.76
**AE**	**5**	**1499.62**	**531**	**437.62**	**ACE**	**0.01**	**1**	**.942**	**.25 [.09, .39]**	**-**	**.75 [.61, .91]**
**CE**	5	1500.49	531	438.49	ACE	0.87	1	.350	-	.19	.81
**E**	4	1509.38	532	445.38	ACE	9.76	2	.008	-	-	1

In bold: the best-fitting model was the non-significant with the lowest AIC.
-2LL = fit statistic, which is minus two times the log-likelihood of the data.
df = degrees of freedom
AIC, fit statistic. Lower values denote better model fits.
Δ χ2 = difference in −2LL statistic between two models, distributed χ2.
Δ *df* = difference in degrees of freedom between two models.

Supplementary Table 13. Bivariate twin model fit statistics for the composite score of face orienting and face preference. Note this is not the same analyses that it was reported in the manuscript (which only included proportion of first look to face). The best fitting model was selected based on non-significance (meaning that there was no decrement in fit compared to the saturated or the genetic model, indexed by the χ2

distribution) and the AIC fit statistic (Akaike information criterion, which incorporates information about both explained variance and parsimoniousness).

Model	# Parameters	-2LL	*df*	AIC	Comparison model	Δ χ2	Δ *df*	P value	A.1	A.12	A.2	C.1	C.12	C.2	E.1	E.12	E.2
**Fully Sat**	32	2845.27	1040	765.27	NA	NA	NA	NA	-	-	-	-	-	-	-	-	-
**5.**	15	2865.95	1057	751.95	Fully Sat	20.68	17	.241	-	-	-	-	-	-	-	-	-
**ACE**	15	2867.28	1057	753.28	Fully Sat.	22.01	17	.184	.22	.41	0	.04	.04	0	.75	.04	.51
**ACE nested models**																	
**AE**	**12**	**2868.67**	**1060**	**748.67**	**ACE**	**1.40**	**3**	**.706**	**.25 [0.09, 0.39]**	**.27 [0.11, 0.53]**	**.19 [0.00, 0.34]**	**-**	**-**	**-**	**.75 [0.61, 0.91]**	**.04 [0.01, 0.09]**	**.51 [0.41, 0.63]**
**CE**	12	2874.65	1060	754.65	ACE	7.37	3	.061	-	-	-	.19	.17	.18	.81	.07	.57
**E**	9	2912.25	1063	786.25	ACE	44.97	6	< .001	-	-	-	-	-	-	1	.18	.82

Model definitions. The Fully Sat. model is the fully saturated model of the observed data, which models the means and variances for both variables, and the phenotypic and cross-twin-cross-trait correlations between the two variables, separately for each twin in a pair and across zygosity. **5.** In the Bivariate model fitting, the constrained saturated model equates means, variances, phenotypic and cross-twin-cross-trait correlations across twins within a pair and across zygosity, for both variables of interest.
In bold: the best-fitting model was the non-significant with the lowest AIC.
-2LL = fit statistic, which is minus two times the log-likelihood of the data.
df = degrees of freedom
AIC, fit statistic. Lower values denote better model fits.
Δ χ2 = difference in −2LL statistic between two models, distributed χ2.
Δ *df* = difference in degrees of freedom between two models.

Supplementary Methods 3. Sensitivity analyses for number of objects explored (in 0-20 seconds)

To fulfil the pre-registered analyses plan, we report here the univariate analyses for number of objects explored operationalized as the number of objects looked at in the whole trial (20 seconds, in contrast with the measure reported in the main manuscript where only the first half of the trial

was included – see more details in the Methods section "Computation of primary measures"). As in the reported measure, the twin correlations suggested no familial influences on the number of objects explored in the entire trial (*ICC* $_{MZ}$=.08, 95% CI [-.10, .25]; *ICC* $_{DZ}$=.12, 95% CI [-.05, .28]). This shows that, the result of the variability in visual exploration being best explained solely by unique environmental factors (which include measurement error) does not seem to be driven by any ceiling effects of the distribution of this measure.

Supplementary Table 14. Descriptive statistics for the number of objects explored during the entire trial length (0-20 seconds). Note this is not the same measure that it is reported in the manuscript (number of objects explored in the first 0-10 seconds).

	**Overall**	**MZ Females**	**MZ Males**	**DZ Females**	**DZ Males**	**Skewness**
**N (twins)**	536	135	158	116	127	
**# Objects explored (in 0-20 seconds)**	4.21 (0.48) 2.50-5.00	4.19 (0.47) 2.67-5.00	4.25 (0.47) 2.83-5.00	4.19 (0.45) 2.67-5.00	4.21 (0.53) 2.50-5.00	-0.84

Supplementary Table 15. Saturated model for the number of objects explored (in 0-20 seconds), including covariates (age and sex). Note this is not the same measure that it is reported in the manuscript (number of objects explored in the first 0-10 seconds). The $\chi 2$ distribution and associated p-value were used to test the effect of the covariates (there was evidence of an effect if there was a significant decrement in fit

compared to the saturated model) and twin model-fitting assumptions (assumptions were violated if there was a significant decrement in fit compared to the saturated model).

					**Comparative fit with saturated model**		
**Model**	**# Parameters**	**-2LL**	***df***	**AIC**	**Δ χ2**	**Δ *df***	**P value**
Saturated	12	722.93	524	-325.07	-	-	-
Age	11	723.35	525	-326.65	0.42	1	.515
Sex	11	724.00	525	-326.00	1.08	1	.300
1.	10	725.47	526	-326.53	2.54	2	.281
2.	9	725.86	527	-328.14	2.94	3	.402
3.	7	726.56	529	-331.44	3.63	5	.604
4.	6	727.13	530	-332.87	4.20	6	.650

Model definitions. The baseline model is the fully saturated model of the observed data, which models the means and variances separately for each twin in a pair and across zygosity. **Age.** Testing the significance of age, **Sex.** Testing the significance of sex, **1.** Equating means across twins within a pair, **2.** Equating means across zygosity, **3.** Equating variances across twins within a pair, and **4.** Equating variances across zygosity (i.e., the constrained saturated model).
-2LL = fit statistic, which is minus two times the log-likelihood of the data.
df = degrees of freedom
AIC, fit statistic. Lower values denote better model fits.
Δ χ2 = difference in −2LL statistic between two models, distributed χ2.
Δ *df* = difference in degrees of freedom between two models.

Supplementary Table 16. Univariate twin model fit statistics and parameter estimates for the number of objects explored (in 0-20 seconds). Note this is not the same measure that it is reported in the manuscript (number of objects explored in the first 0-10 seconds). The best fitting model was selected based on non-significance (meaning that there was no decrement in fit compared to the saturated or the genetic model, indexed by

the χ2 distribution) and the AIC fit statistic (Akaike information criterion, which incorporates information about both explained variance and parsimoniousness).

Model	# Parameters	-2LL	*df*	AIC	Comparison model	Δ χ2	Δ *df*	P value	A	C	E
**Fully Sat**	12	722.93	524	-325.07	NA	-	-	-	-	-	-
**ACE**	6	727.24	530	-332.76	Fully Sat.	4.32	6	.634	0	.10	.90
**AE**	5	727.82	531	-334.18	ACE	0.57	1	.449	.11	-	.89
**CE**	**5**	**727.24**	**531**	**-334.76**	**ACE**	**0.00**	**1**	**> .999**	**-**	**.10 [0, .22]**	**.90 [.78, 1]**
**E**	4	729.80	532	-334.20	ACE	2.55	2	.279	-	-	1

In bold: the best-fitting model was the non-significant with the lowest AIC.
-2LL = fit statistic, which is minus two times the log-likelihood of the data.
df = degrees of freedom
AIC, fit statistic. Lower values denote better model fits.
Δ χ2 = difference in −2LL statistic between two models, distributed χ2.
Δ *df* = difference in degrees of freedom between two models

Supplementary Methods 4. Analyses for non-social attention

During the peer-reviewed process, it was noted that, in order to understand if effects in face orienting and preference (which reflected social versus non-social preferences) were indeed specific to social/face objects, and not generalized to the most attended object in a scene, these could be contrasted to orienting and preference to the most attended non-social object in the pop-out arrays. Specifically, after face (looked at 44% of time), the most looked at object was the car (19% of time). Therefore, car orienting/ first looks to the car was calculated relative to all other non-social objects (i.e., excluding the trials where face was looked at first), as well as looking preference for the car relative to all non-social objects

(i.e., excluding looking time to the face). These measures can be seen as approximate analogies to the reported face attention variables, while at the same time are mathematical independent of these variables.

Car preference (Mean proportion = .34) and car orienting (Mean proportion = .33) were significantly above the chance level (which is .25 because we excluded the face). There were no significant genetic effects in terms of either car orienting (E model had the lowest AIC) or car preference (the univariate AE model had the lowest AIC but the genetic effect estimate was not significant, see table below). In terms of the bivariate model between car orienting and car preference (rPh = .25, 95% CI [0.17, 0.33]), the E model had the lowest AIC, with most E on car preference being unique to that variable (unique E = 0.94, 95% CI [0.89, 0.97]), and just a small significant proportion being shared with car orienting (shared E = 0.06, 95% CI [0.03, 0.11]). These analyses suggest that while social (face) preference and orienting has a clear genetic contribution, etiological influences to preference and orienting to the second most salient object (car) are different, and do not seem to include substantial familial effects.

Supplementary Table 17. Twin correlation coefficients (95% confidence intervals are shown in parentheses) for the primary face looking measures, separate for MZ and DZ pairs.

	N (Twin pairs*)	Car orienting	Preference for car
**MZ**	155	-.03 [-.21, .16]	.21 [.05, .36]
**DZ**	130	.03 [-.13, .19]	-.11 [-.28, .07]

** Incomplete twin pairs*
Correlations were derived from the twin models with age and sex included as covariates.

Supplementary Table 18. Descriptive statistics for car orienting and preference.

	Overall	MZ Females	MZ Males	DZ Females	DZ Males	Skewness
**N (twins)**	536	135	158	116	127	
**Car orienting**	0.33 (0.25)<br>0-1	0.34 (0.26)<br>0-1	0.32 (0.23)<br>0-1	0.33 (0.27)<br>0-1	0.34 (0.26)<br>0-1	0.37
**Car preference**	0.34 (0.11)<br>0.07-0.76	0.33 (0.11)<br>0.09-0.64	0.34 (0.1)<br>0.07-0.62	0.34 (0.11)<br>0.13-0.76	0.34 (0.1)<br>0.09-0.7	0.30

Supplementary Table 19. Saturated model for car orienting (proportion of first look to car) and car preference, including covariates (age and sex). The χ2 distribution and associated p-value were used to test the effect of the covariates (there was evidence of an effect if there was a significant decrement in fit compared to the saturated model) and twin model-fitting assumptions (assumptions were violated if there was a significant decrement in fit compared to the saturated model).

					**Comparative fit with saturated model**		
**Model**	**# Parameters**	**-2LL**	***df***	**AIC**	**Δ χ2**	**Δ *df***	**P value**
**Car orienting**							
Saturated	12	1484.49	524	436.49	-	-	-
Age	11	1514.21	524	466.21	2.19	1	0.139
Sex	11	1512.16	524	464.16	0.14	1	0.711
1.	10	1512.05	525	462.05	0.02	2	0.989
2.	9	1512.07	526	460.07	0.05	3	0.997
3.	7	1512.11	528	456.11	0.09	5	1

4.	6	1514.73	529	456.73	2.70	6	0.845
**Car preference**							
Saturated	12	-876.71	524	-1924.71	-	-	-
Age	11	-875.64	525	-1925.64	1.07	1	0.300
Sex	11	-875.91	525	-1925.91	0.81	1	0.369
1.	10	-876.25	526	-1928.25	0.46	2	0.793
2.	9	-875.71	527	-1929.71	1.00	3	0.800
3.	7	-870.97	529	-1928.97	5.74	5	0.332
4.	6	-870.97	530	-1930.97	5.75	6	0.452

Model definitions. The baseline model is the fully saturated model of the observed data, which models the means and variances separately for each twin in a pair and across zygosity. **Age.** Testing the significance of age, **Sex.** Testing the significance of sex, **1.** Equating means across twins within a pair, **2.** Equating means across zygosity, **3.** Equating variances across twins within a pair, and **4.** Equating variances across zygosity (i.e., the constrained saturated model).
-2LL = fit statistic, which is minus two times the log-likelihood of the data.
df = degrees of freedom
AIC, fit statistic. Lower values denote better model fits.
Δ χ2 = difference in −2LL statistic between two models, distributed χ2.
Δ *df* = difference in degrees of freedom between two models.

Supplementary Table 20. Univariate twin model fit statistics and parameter estimates for car orienting (proportion of first look to car) and car preference, including covariates. The best fitting model was selected based on non-significance (meaning that there was no decrement in fit compared to the saturated or the genetic model, indexed by the χ2 distribution) and the AIC fit statistic (Akaike information criterion, which incorporates information about both explained variance and parsimoniousness).

**Model**	**# parameters**	**-2LL**	***df***	**AIC**	**Comparison model**	**Δ χ2**	**Δ *df***	**P value**	**A**	**C**	**E**
**Car orienting**											
**Fully Sat**	12	1512.03	523	466.03	NA	-	-	-	-	-	-

**ACE**	6	1514.91	529	456.91	Fully Sat.	2.89	6	0.823	0	<.01	>.99
**AE**	5	1514.92	530	454.92	ACE	<0.01	1	0.953	0	-	1
**CE**	5	1514.91	530	454.91	ACE	<0.01	1	1	-	<.01	>.99
**E**	**4**	**1514.92**	**531**	**452.92**	**ACE**	**<0.01**	**2**	**0.998**	**-**	**-**	**1**
**Car preference**											
**Fully Sat**	12	-876.71	524	-1924.71	NA	-	-	-	-	-	-
**ACE**	6	-866.55	530	-1926.55	Fully Sat.	10.16	6	0.118	.14	0	.86
**AE**	**5**	**-866.55**	**531**	**-1928.55**	**ACE**	**<0.01**	**1**	**1**	**.14 [0, .29]**	**-**	**.86 [.71, 1]**
**CE**	5	-864.53	531	-1926.53	ACE	2.02	1	0.156	-	.06	.94
**E**	4	-863.53	532	-1927.53	ACE	3.01	2	0.222	-	-	1

In bold: the best-fitting model was the non-significant with the lowest AIC.
-2LL = fit statistic, which is minus two times the log-likelihood of the data.
df = degrees of freedom
AIC, fit statistic. Lower values denote better model fits.
Δ χ2 = difference in −2LL statistic between two models, distributed χ2.
Δ *df* = difference in degrees of freedom between two models.

Supplementary Table 21. Bivariate twin model fit statistics for car orienting and car preference. The best fitting model was selected based on non-significance (meaning that there was no decrement in fit compared to the saturated or the genetic model, indexed by the χ2 distribution) and the AIC fit statistic (Akaike information criterion, which incorporates information about both explained variance and parsimoniousness).

Model	# Parameters	-2LL	*df*	AIC	Comparison model	Δ χ2	Δ *df*	P value	A.1	A.12	A.2	C.1	C.12	C.2	E.1	E.12	E.2
**Fully Sat**	32	593.69	1039	-1484.31	NA	NA	NA	NA	-	-	-	-	-	-	-	-	-
**5.**	15	608.08	1056	-1503.92	Fully Sat	14.39	17	0.639	-	-	-	-	-	-	-	-	-
**ACE**	15	613.41	1056	-1498.59	Fully Sat.	19.72	17	0.289	0.01	0.14	0	0	0	0	0.99	0.05	0.81

**ACE nested models**																	
**AE**	12	613.41	1059	-1504.59	ACE	<0.01	3	1	0.01	0.14	0	-	-	-	0.99	0.05	0.81
**CE**	12	615.43	1059	-1502.57	ACE	2.02	3	0.567	-	-	-	0.01	0.06	0	0.99	0.05	0.88
**E**	**9**	**616.44**	**1062**	**-1507.56**	**ACE**	**3.03**	**6**	**0.805**	-	-	-	-	-	-	**1**	**.06 [.03, .11]**	**.94 [.89, .97]**

Model definitions. The Fully Sat. model is the fully saturated model of the observed data, which models the means and variances for both variables, and the phenotypic and cross-twin-cross-trait correlations between the two variables, separately for each twin in a pair and across zygosity. **5.** In the Bivariate model fitting, the constrained saturated model equates means, variances, phenotypic and cross-twin-cross-trait correlations across twins within a pair and across zygosity, for both variables of interest.
In bold: the best-fitting model was the non-significant with the lowest AIC.
-2LL = fit statistic, which is minus two times the log-likelihood of the data.
df = degrees of freedom
AIC, fit statistic. Lower values denote better model fits.
Δ $\chi 2$ = difference in −2LL statistic between two models, distributed $\chi 2$.
Δ *df* = difference in degrees of freedom between two models.

Supplementary Methods 5. Methods for the computation of genome-wide polygenic scores

Quality control and Imputation

The array consists of 730 059 markers of which 97.8% had a sample call rate 98% with average SNP call rate per sample 99.3%. We performed both quality control (QC) for the raw genotyping and imputed calls at the individual and marker level using PLINK v1.90. Both autosomal and X chromosomes were imputed using IMPUTE2. The following QC criteria was used to identify the outlier individuals and remove the low-quality markers:individual QC: discordant sex, heterozygosity rate > 3SD, individual genotype failure rate > 0.03, and relatedness; marker QC: info score < 0.8, minor allele frequency < 0.01, Hardy-Weinberg equilibrium < 1e-06, individual missingness < 0.1, and marker missingness < 0.05. There were 518 570 markers remained after genotyping QC and 7 507 876 markers after imputation QC.

Genetic ancestry

To investigate the genetic ancestry of the twins, we compared the genotyping information to two resources of reference data HapMap Phase III (HapMap3) and SWEGEN [1, 2]. A principal component analysis (PCA) by EIGENSOFT 7.2.1 was performed and the largest two components were used to illustrate the genetic ancestry using R 3.6.3.

Supplementary Methods 6. Power analysis

The current paper describes results that were part of a larger project (BATSS), which consists of several experiments and other types of assessments. We conducted a general, broad scope power analysis prior to data collection in BATSS, in which we assumed 225 pairs would have valid data, and a MZ/DZ ratio of 0.5. A study of this size has nearly 97% power to detect a heritability of 40% and a shared environmental effect of 40% for a phenotype. We will have nearly 88% power to detect a significant genetic contribution to a correlation between two measures, assuming that the heritability of the two variables is 40%, that the shared environment explains 40% of the variation in the two variables, and that the phenotypic correlation is $r = 0.40$ and to the same degree mediated by genetic and shared environmental effects.

Supplementary Methods 7. Sensitivity Analyses for additional gaze quality metrics

During the peer-reviewed process, it was noted that additional gaze quality covariates (accuracy and precision) should be tested for effects on the gaze-based primary visual attention measures. We used accuracy (drift in Euclidean distance from center of stimuli and mean point of gaze) and

precision (root mean square of the gaze inter-sample Euclidean distances) measured in four post-hoc calibration stimuli presented in randomized positions at fixed time points during the task battery. Associations between accuracy and precision, and the visual attention measures, were tested within the GEE framework (one linear model with all gaze quality covariates as predictors were run for each primary variable).

Accuracy was found to be significantly related to face preference (in addition to proportion of missing gaze), no other links were found.

Accuracy and proportion of missing data were regressed from face preference before repeating analyses. Results were similar.

Results were the same as before for the univariate twin modelling of face preference, for the bivariate twin modelling (face preference and face orienting), and for the associations with questionnaire data and with polygenic scores.

Supplementary Table 22. Univariate saturated model for face preference (proportion looking time on face, regressed on proportion of missing gaze and accuracy) including covariates (age and sex). The χ2 distribution and associated p-value were used to test the effect of the covariates (there was evidence of an effect if there was a significant decrement in fit compared to the saturated model) and twin model-fitting assumptions (assumptions were violated if there was a significant decrement in fit compared to the saturated model).

					**Comparative fit with saturated model**		
**Model**	**# Parameters**	**-2LL**	***df***	**AIC**	**Δ χ2**	**Δ *df***	**P value**
Saturated	12	1465.21	524	417.21	-	-	-
**Age**	**11**	**1471.64**	**525**	**421.64**	**6.42**	**1**	**.011**
Sex	11	1467.94	525	417.94	2.73	1	.099
1.	10	1467.61	526	415.61	2.40	2	.301
2.	9	1467.64	527	413.64	2.43	3	.488

3.	7	1469.32	529	411.32	4.10	5	.535
4.	6	1470.46	530	410.46	5.25	6	.513

Model definitions. The baseline model is the fully saturated model of the observed data, which models the means and variances separately for each twin in a pair and across zygosity. **Age.** Testing the significance of age, **Sex.** Testing the significance of sex, **1.** Equating means across twins within a pair, **2.** Equating means across zygosity, **3.** Equating variances across twins within a pair, and **4.** Equating variances across zygosity (i.e., the constrained saturated model).
In bold: models with a significant poorer fit compared with the saturated model.
-2LL = fit statistic, which is minus two times the log-likelihood of the data.
df = degrees of freedom
AIC, fit statistic. Lower values denote better model fits.
Δ χ2 = difference in −2LL statistic between two models, distributed χ2.
Δ *df* = difference in degrees of freedom between two models.

Supplementary Table 23. Univariate twin model fit statistics and parameter estimates for face preference (proportion looking time on face, regressed on proportion of missing gaze and accuracy). The best fitting model was selected based on non-significance (meaning that there was no decrement in fit compared to the saturated or the genetic model, indexed by the χ2 distribution) and the AIC fit statistic (Akaike information criterion, which incorporates information about both explained variance and parsimoniousness).

**Model**	**# parameters**	**-2LL**	***df***	**AIC**	**Comparison model**	**Δ χ2**	**Δ *df***	**P value**	**A**	**C/D**	**E**
**Fully Sat**	12	1465.21	524	417.21	NA	-	-	-	-	-	-
**ACE**	6	1470.73	530	410.73	Fully Sat.	5.51	6	.480	.45	0	.55
**AE**	**5**	**1470.73**	**531**	**408.73**	**ACE**	**0.00**	**1**	**> .999**	**.45 [.32, .56]**	**-**	**.55 [.44, .68]**
**CE**	5	1476.99	531	414.99	ACE	6.26	1	.012	-	.35	.65
**E**	4	1508.04	532	444.04	ACE	37.31	2	< .001	-	-	1

In bold: the best-fitting model was the non-significant with the lowest AIC.
-2LL = fit statistic, which is minus two times the log-likelihood of the data.
df = degrees of freedom
AIC, fit statistic. Lower values denote better model fits.
Δ χ2 = difference in −2LL statistic between two models, distributed χ2.

Δ *df* = difference in degrees of freedom between two models.

Supplementary Table 24. Assumption testing for the bivariate model between face orienting (proportion of first look to faces) and face preference (regressed on proportion of missing gaze and accuracy). The χ2 distribution and associated p-value was used to test the twin model-fitting assumptions (assumptions were violated if there was a significant decrement in fit compared to the saturated model).

					**Comparative fit with saturated model**		
**Model**	**# Parameters**	**-2LL**	***df***	**AIC**	**Δ χ2**	**Δ *df***	**P value**
Saturated	32	1153.28	1040	-926.72	-	-	-
5.	15	1167.28	1057	-946.72	14.00	17	.667

Model definitions. The Fully Sat. model is the fully saturated model of the observed data, which models the means and variances for both variables, and the phenotypic and cross-twin-cross-trait correlations between the two variables, separately for each twin in a pair and across zygosity. **5.** In the Bivariate model fitting, the constrained saturated model equates means, variances, phenotypic and cross-twin-cross-trait correlations across twins within a pair and across zygosity, for both variables of interest.
-2LL = fit statistic, which is minus two times the log-likelihood of the data.
df = degrees of freedom
AIC, fit statistic. Lower values denote better model fits.
Δ χ2 = difference in −2LL statistic between two models, distributed χ2.
Δ *df* = difference in degrees of freedom between two models.

Supplementary Table 25. ACE Bivariate twin model fit statistics and estimates between face orienting (proportion of first look to faces) and face preference (regressed on proportion of missing gaze and accuracy). Best-fitting model in bold. The best fitting model was selected based on non-

significance (meaning that there was no decrement in fit compared to the saturated or the genetic model, indexed by the χ2 distribution) and the AIC fit statistic (Akaike information criterion, which incorporates information about both explained variance and parsimoniousness).

Model	# Parameters	-2LL	*df*	AIC	Comparison model	Δ χ2	Δ *df*	P value	A.1	A.12	A.2	C.1	C.12	C.2	E.1	E.12	E.2
**Fully Sat**	32	1153.28	1040	-926.72	NA	NA	NA	NA	-	-	-	-	-	-	-	-	-
**ACE**	15	1169.63	1057	-944.37	Fully Sat.	16.35	17	.499	.14	.38	0	.05	.07	0	.81	.02	.53
**AE**	**12**	**1170.90**	**1060**	**-949.10**	**ACE**	**1.27**	**3**	**.735**	**.19 [0.04, 0.33]**	**.15 [0.03, 0.50]**	**.30 [0, 0.45]**	**-**	**-**	**-**	**.81 [0.67, 0.96]**	**.02 [0, 0.06]**	**.53 [0.42, 0.65]**
**CE**	12	1177.75	1060	-942.25	ACE	8.12	3	.044	-	-	-	.13	.08	.27	.87	.04	.61
**E**	9	1212.04	1063	-913.96	ACE	42.41	6	<.001	-	-	-	-	-	-	1	.09	.91

Model definitions. The Fully Sat. model is the fully saturated model of the observed data, which models the means and variances for both variables, and the phenotypic and cross-twin-cross-trait correlations between the two variables, separately for each twin in a pair and across zygosity.
In bold: the best-fitting model was the non-significant with the lowest AIC.
-2LL = fit statistic, which is minus two times the log-likelihood of the data.
df = degrees of freedom
AIC, fit statistic. Lower values denote better model fits.
Δ χ2 = difference in −2LL statistic between two models, distributed χ2.
Δ *df* = difference in degrees of freedom between two models.
A = additive genetic influences
C = shared environment influences
E = non-shared environment influences
A-C-E.1 = variance on Phenotype 1 (proportion of first look to face)
A-C-E.12 = variance on Phenotype 2 (face preference) that is shared with Phenotype 1 (proportion of first look to face)
A-C-E.2 = unique variance on Phenotype 2 (face preference)

Supplementary Table 26. Results of multiple Generalized Estimating Equations analyses with 14 months and 24 months parent-report measures as outcome variables, age, sex, and the looking measures measured at 5 months as predictors, and twin pair id as cluster-defining variable. For

each model, all predictors were entered together; hence statistics represent unique contributions for each predictor. Adjustments were made for multiple comparisons using the False Discovery Rate step-up approach.

**ECBQ Self-regulation at 14 months**	**Beta**	**SE**	**95% CI**	**Uncorrected *p*-value**	**FDR Threshold**
Age (in days, scaled)	-0.08	0.06	[-0.19, 0.03]	0.160	0.01
Sex (reference level: Female)	0.06	0.12	[-0.17, 0.29]	0.605	0.04
Face orienting (proportion first look to the face)	0.07	0.05	[-0.04, 0.17]	0.212	0.02
Preference for face (proportion on face)	0.00	0.06	[-0.11, 0.11]	0.975	0.05
# Objects explored (in 0-10 seconds)	0.04	0.05	[-0.06, 0.14]	0.430	0.03
**ECBQ Self-regulation at 24 months**	**Beta**	**SE**	**95% CI**	**Uncorrected *p*-value**	**FDR Threshold**
Age (in days, scaled)	0.05	0.07	[-0.09, 0.19]	0.468	0.04
**Sex (reference level: Female)**	**-0.38**	**0.13**	**[-0.64, -0.13]**	**0.003**	**0.01**
Face orienting (proportion first look to the face)	0.06	0.06	[-0.05, 0.17]	0.259	0.02
Preference for face (proportion on face)	0.04	0.06	[-0.07, 0.16]	0.477	0.05
# Objects explored (in 0-10 seconds)	0.04	0.05	[-0.06, 0.14]	0.430	0.03
**ITC Social communication at 14 months**	**Beta**	**SE**	**95% CI**	**Uncorrected *p*-value**	**FDR Threshold**
Age (in days, scaled)	0.07	0.07	[-0.07, 0.21]	0.344	.04
Sex (reference level: Female)	-0.27	0.12	[-0.51, -0.03]	0.030	.02
Face orienting (proportion first look to the face)	-0.01	0.06	[-0.12, 0.11]	0.921	.05
Preference for face (proportion on face)	0.12	0.06	[0.01, 0.23]	0.030	.01
# Objects explored (in 0-10 seconds)	0.10	0.05	[0.00, 0.19]	0.043	.03
**CDI Receptive vocabulary at 14 months**	**Beta**	**SE**	**95% CI**	**Uncorrected *p*-value**	**FDR Threshold**
**Age (in days, scaled)**	**0.15**	**0.06**	**[0.04, 0.27]**	**0.008**	**.01**
**Sex (reference level: Female)**	**-0.34**	**0.13**	**[-0.59, -0.09]**	**0.008**	**.02**
Face orienting (proportion first look to the face)	-0.01	0.05	[-0.11, 0.09]	0.822	.05
**Preference for face (proportion on face)**	**0.14**	**0.06**	**[0.03, 0.25]**	**0.016**	**.03**
# Objects explored (in 0-10 seconds)	0.06	0.05	[-0.03, 0.16]	0.184	.04
**CDI Expressive vocabulary at 24 months**	**Beta**	**SE**	**95% CI**	**Uncorrected *p*-value**	**FDR Threshold**
**Age (in days, scaled)**	**0.21**	**0.05**	**[0.11, 0.31]**	**< .001**	**.02**
**Sex (reference level: Female)**	**-0.58**	**0.14**	**[-0.84, -0.31]**	**< .001**	**.01**
Face orienting (proportion first look to the face)	-0.01	0.05	[-0.11, 0.10]	0.905	.05
Preference for face (proportion on face)	0.03	0.06	[-0.09, 0.14]	0.674	.04
# Objects explored (in 0-10 seconds)	0.09	0.05	[-0.00, 0.19]	0.062	.03

In bold: significant predictors (see p-threshold in the False Discovery Rate threshold column).

Supplementary Table 27. Summary of the two Generalized Estimating Equations models including genome-wide polygenic scores (GPSs) for autism, ADHD, bipolar disorder, major depressive disorder, and schizophrenia, 10 principal component of ancestry, and age and sex, as predictors of face preference (regressed on proportion of missing gaze and accuracy), with twin pair id as cluster-defining variable. Significant predictors are in bold (adjustments were made for multiple comparisons setting the alpha threshold for the number of outcomes, = .025).

Face preference (proportion on face)	Beta	95% CI	Uncorrected *p*-value
**Age (in days)**	**0.01**	**[0.00, 0.02]**	**0.009**
Sex	-0.16	[-0.35, 0.02]	0.089
GPS autism	-0.03	[-0.15, 0.08]	0.594
GPS ADHD	0.00	[-0.11, 0.11]	0.952
GPS bipolar disorder	-0.04	[-0.20, 0.12]	0.625
GPS major depression disorder	0.05	[-0.06, 0.15]	0.400
GPS schizophrenia	0.06	[-0.07, 0.19]	0.368
PC 2	-14.16	[-29.04, 0.72]	0.062
PC 3	-0.24	[-11.20, 10.73]	0.966
PC 4	-23.17	[-46.45, 0.11]	0.051
PC 5	-12.38	[-34.26, 9.50]	0.267
PC 6	-6.89	[-26.09, 12.30]	0.482
PC 7	-9.31	[-29.14, 10.53]	0.358
PC 8	5.76	[-13.20, 24.72]	0.552
PC 9	20.11	[-0.30, 40.53]	0.053

PC 10	4.35	[-16.90, 25.61]	0.688
PC 11	16.19	[-2.97, 35.34]	0.098

In bold: significant predictors.

Supplementary Methods 8. Sensitivity Analyses for corrected age

During the peer-reviewed process, it was noted that the corrected age (age estimated based on birth date and gestational age at birth) of infants, rather than their age calculated based solely on their birth date (chronological age), should be included as a covariate in the analyses as a sensitivity check, due to the increased risk of premature births in twin samples. Corrected age was calculated based on chronological age in days minus the number of days early they were born.

Results were the same as with age based on birth for the univariate twin modelling of face preference, for the bivariate twin modelling (face preference and face orienting), and for the associations with polygenic scores. For the longitudinal phenotypic associations with developmental outcomes, the statistically significant positive association between preference for the face and receptive vocabulary (comprehension in the CDI) at 14 months was no longer significant, but this was marginal, $p = 0.034$, threshold = 0.03.

Supplementary Table 28. Univariate saturated model for the number of objects explored (in 0-10 seconds) including covariates (corrected age and sex). The $\chi2$ distribution and associated p-value were used to test the effect of the covariates (there was evidence of an effect if there was a

significant decrement in fit compared to the saturated model) and twin model-fitting assumptions (assumptions were violated if there was a significant decrement in fit compared to the saturated model).

					Comparative fit with saturated model		
**Model**	**# Parameters**	**-2LL**	***df***	**AIC**	**Δ χ2**	**Δ *df***	**P value**
Saturated	12	1507.06	524	459.06	-	-	-
Age	11	1507.21	525	457.21	0.15	1	0.696
Sex	11	1508.95	525	458.95	1.89	1	0.169
1.	10	1512.38	526	460.38	5.32	2	0.070
2.	9	1512.40	527	458.40	5.34	3	0.149
3.	7	1514.37	529	456.37	7.31	5	0.198
4.	6	1514.86	530	454.86	7.80	6	0.253

Model definitions. The baseline model is the fully saturated model of the observed data, which models the means and variances separately for each twin in a pair and across zygosity. **Age.** Testing the significance of age, **Sex.** Testing the significance of sex, **1.** Equating means across twins within a pair, **2.** Equating means across zygosity, **3.** Equating variances across twins within a pair, and **4.** Equating variances across zygosity (i.e., the constrained saturated model).
-2LL = fit statistic, which is minus two times the log-likelihood of the data.
df = degrees of freedom
AIC, fit statistic. Lower values denote better model fits.
Δ χ2 = difference in −2LL statistic between two models, distributed χ2.
Δ *df* = difference in degrees of freedom between two models.

Supplementary Table 29. Univariate twin model fit statistics and parameter estimates for number of objects explored (in 0-10 seconds) including covariates (corrected age and sex). The best fitting model was selected based on non-significance (meaning that there was no decrement in fit

compared to the saturated or the genetic model, indexed by the χ2 distribution) and the AIC fit statistic (Akaike information criterion, which incorporates information about both explained variance and parsimoniousness).

Model	# parameters	-2LL	*df*	AIC	Comparison model	Δ χ2	Δ *df*	P value	A	C	E
**Fully Sat**	12	1507.06	524	459.06	NA	-	-	-	-	-	-
**ACE**	6	1515.48	530	455.48	Fully Sat.	8.42	6	0.209	0	.10	.90
**AE**	5	1516.43	531	454.43	ACE	0.95	1	.329	.10	-	.90
**CE**	**5**	**1515.48**	**531**	**453.48**	**ACE**	**0.00**	**1**	**> .999**	**-**	**.10 [0, .22]**	**.90 [.78, 1]**
**E**	4	1518.03	532	454.03	ACE	2.55	2	.279	-	-	1

In bold: the best-fitting model was the non-significant with the lowest AIC.
-2LL = fit statistic, which is minus two times the log-likelihood of the data.
df = degrees of freedom
AIC, fit statistic. Lower values denote better model fits.
Δ χ2 = difference in −2LL statistic between two models, distributed χ2.
Δ *df* = difference in degrees of freedom between two models.

Supplementary Table 30. Assumption testing for the bivariate model between face orienting (proportion of first look to faces) and face preference, including covariates (corrected age and sex). The χ2 distribution and associated p-value was used to test the twin model-fitting assumptions (assumptions were violated if there was a significant decrement in fit compared to the saturated model).

					Comparative fit with saturated model		
**Model**	**# Parameters**	**-2LL**	***df***	**AIC**	**Δ χ2**	**Δ *df***	**P value**
Saturated	32	1136.97	1040	-943.03	-	-	-
5.	15	1150.59	1057	-963.41	13.62	17	0.693

Model definitions. The Fully Sat. model is the fully saturated model of the observed data, which models the means and variances for both variables, and the phenotypic and cross-twin-cross-trait correlations between the two variables, separately for each twin in a pair and across zygosity. **5.** In the Bivariate model fitting,

the constrained saturated model equates means, variances, phenotypic and cross-twin-cross-trait correlations across twins within a pair and across zygosity, for both variables of interest.
The best-fitting model (in bold) was the non-significant and most parsimonious model, as well as the one with the lowest AIC.
-2LL = fit statistic, which is minus two times the log-likelihood of the data.
df = degrees of freedom
AIC, fit statistic. Lower values denote better model fits.
Δ $\chi2$ = difference in −2LL statistic between two models, distributed $\chi2$.
Δ *df* = difference in degrees of freedom between two models.

Supplementary Table 31. ACE Bivariate twin model fit statistics and estimates between face orienting (proportion of first look to faces) and face preference, including covariates (corrected age and sex). The best fitting model was selected based on non-significance (meaning that there was no decrement in fit compared to the saturated or the genetic model, indexed by the $\chi2$ distribution) and the AIC fit statistic (Akaike information criterion, which incorporates information about both explained variance and parsimoniousness).

Model	# Parameters	-2LL	*df*	AIC	Comparison model	Δ $\chi2$	Δ *df*	P value	A.1	A.12	A.2	C.1	C.12	C.2	E.1	E.12	E.2
**Fully Sat**	32	1136.97	1040	-943.03	NA	NA	NA	NA	-	-	-	-	-	-	-	-	-
**ACE**	15	1153.12	1057	-960.88	Fully Sat.	16.15	17	0.513	.14	.34	0	.04	.08	0	.82	.02	.56
**AE**	**15**	**1154.70**	**1060**	**-965.30**	**ACE**	**1.58**	**3**	**.664**	**.18 [0.03, 0.32]**	**.14 [0.02, 0.49]**	**.29 [0, 0.44]**	**-**	**-**	**-**	**.82 [0.68, 0.97]**	**.02 [0, 0.06]**	**.55 [0.44, 0.68]**
**CE**	12	1160.18	1060	-959.82	ACE	7.05	3	.070	-	-	-	.13	.07	.26	.88	.04	.63
**E**	9	1191.65	1063	-934.35	ACE	38.53	6	< .001	-	-	-	-	-	-	1	.08	.92

Model definitions. The Fully Sat. model is the fully saturated model of the observed data, which models the means and variances for both variables, and the phenotypic and cross-twin-cross-trait correlations between the two variables, separately for each twin in a pair and across zygosity.
In bold: the best-fitting model was the non-significant with the lowest AIC.
-2LL = fit statistic, which is minus two times the log-likelihood of the data.
df = degrees of freedom
AIC, fit statistic. Lower values denote better model fits.
Δ $\chi2$ = difference in −2LL statistic between two models, distributed $\chi2$.

Δ *df* = difference in degrees of freedom between two models.
A = additive genetic influences
C = shared environment influences
E = non-shared environment influences
A-C-E.1 = variance on Phenotype 1 (proportion of first look to face)
A-C-E.12 = variance on Phenotype 2 (face preference) that is shared with Phenotype 1 (proportion of first look to face)
A-C-E.2 = unique variance on Phenotype 2 (face preference)

Supplementary Table 32. Results of multiple Generalized Estimating Equations analyses with 14 months and 24 months parent-report measures as outcome variables, age, sex, and the looking measures measured at 5 months as predictors, and twin pair id as cluster-defining variable. For each model, all predictors were entered together; hence statistics represent unique contributions for each predictor. Adjustments were made for multiple comparisons using the False Discovery Rate step-up approach.

**ECBQ Self-regulation at 14 months**	**Beta**	**SE**	**95% CI**	**Uncorrected *p*-value**	**FDR Threshold**
Age (in days, scaled)	-0.08	0.06	[-0.19, 0.03]	0.158	0.01
Sex (reference level: Female)	0.06	0.12	[-0.17, 0.29]	0.610	0.04
Face orienting (proportion first look to the face)	0.06	0.05	[-0.04, 0.17]	0.232	0.02
Preference for face (proportion on face)	-0.01	0.05	[-0.11, 0.10]	0.921	0.05
# Objects explored (in 0-10 seconds)	0.04	0.05	[-0.06, 0.14]	0.412	0.03
**ECBQ Self-regulation at 24 months**	**Beta**	**SE**	**95% CI**	**Uncorrected *p*-value**	**FDR Threshold**
Age (in days, scaled)	0.05	0.07	[-0.09, 0.19]	0.473	0.03
**Sex (reference level: Female)**	**-0.38**	**0.13**	**[-0.63, -0.13]**	**0.003**	**0.01**
Face orienting (proportion first look to the face)	0.06	0.06	[-0.05, 0.17]	0.252	0.02
Preference for face (proportion on face)	0.03	0.06	[-0.09, 0.15]	0.610	0.05
# Objects explored (in 0-10 seconds)	0.03	0.05	[-0.07, 0.14]	0.530	0.04
**ITC Social communication at 14 months**	**Beta**	**SE**	**95% CI**	**Uncorrected *p*-value**	**FDR Threshold**
Age (in days, scaled)	0.07	0.07	[-0.07, 0.21]	0.344	0.04
Sex (reference level: Female)	-0.27	0.12	[-0.51, -0.03]	0.030	0.03
Face orienting (proportion first look to the face)	0.00	0.06	[-0.12, 0.11]	0.938	0.05
Preference for face (proportion on face)	0.13	0.05	[0.02, 0.24]	0.019	0.01

# Objects explored (in 0-10 seconds)	0.10	0.05	[0.01, 0.20]	0.027	0.02
**CDI Receptive vocabulary at 14 months**	**Beta**	**SE**	**95% CI**	**Uncorrected *p*-value**	**FDR Threshold**
**Age (in days, scaled)**	**0.15**	**0.06**	**[0.04, 0.27]**	**0.008**	**.01**
**Sex (reference level: Female)**	**-0.34**	**0.13**	**[-0.60, -0.09]**	**0.008**	**.02**
Face orienting (proportion first look to the face)	-0.02	0.05	[-0.11, 0.08]	0.756	.05
Preference for face (proportion on face)	0.12	0.06	[0.01, 0.23]	0.034	.03
# Objects explored (in 0-10 seconds)	0.06	0.05	[-0.03, 0.16]	0.175	.04
**CDI Expressive vocabulary at 24 months**	**Beta**	**SE**	**95% CI**	**Uncorrected *p*-value**	**FDR Threshold**
**Age (in days, scaled)**	**0.21**	**0.05**	**[0.11, 0.31]**	**< .001**	**.02**
**Sex (reference level: Female)**	**-0.58**	**0.14**	**[-0.85, -0.32]**	**< .001**	**.01**
Face orienting (proportion first look to the face)	-0.01	0.05	[-0.11, 0.09]	0.869	.05
Preference for face (proportion on face)	0.03	0.06	[-0.08, 0.15]	0.581	.04
# Objects explored (in 0-10 seconds)	0.10	0.05	[0.00, 0.20]	0.043	.03

In bold: significant predictors (see p-threshold in the False Discovery Rate threshold column).

Supplementary Table 33. Summary of the two Generalized Estimating Equations models including genome-wide polygenic scores (GPSs) for autism, ADHD, bipolar disorder, major depressive disorder, and schizophrenia, 10 principal component of ancestry, and corrected age and sex, as predictors of face orienting (proportion of first look to faces) or face preference, with twin pair id as cluster-defining variable. Significant predictors are in bold (adjustments were made for multiple comparisons setting the alpha threshold for the number of outcomes, = .025).

**Face orienting (Proportion first look to face)**	**Beta**	**95% CI**	**Uncorrected *p*-value**
**Corrected age (in days)**	**0.02**	**[0.01, 0.04]**	**0.008**
Sex	-0.03	[-0.06, 0.00]	0.081
GPS autism	-0.01	[-0.03, 0.01]	0.545
GPS ADHD	-0.01	[-0.03, 0.01]	0.237
GPS bipolar disorder	0.00	[-0.02, 0.03]	0.834

GPS major depression disorder	0.02	[-0.00, 0.04]	0.054
GPS schizophrenia	-0.02	[-0.05, 0.00]	0.051
PC 2	0.30	[-2.99, 3.58]	0.860
**PC 3**	**-1.95**	**[-3.63, -0.28]**	**0.022**
PC 4	2.85	[-0.67, 6.36]	0.113
PC 5	3.47	[-0.35, 7.30]	0.075
PC 6	-0.05	[-3.63, 3.54]	0.980
PC 7	-0.63	[-3.80, 2.54]	0.698
PC 8	-0.63	[-3.74, 2.48]	0.690
PC 9	3.33	[-0.34, 7.01]	0.075
PC 10	0.09	[-2.99, 3.16]	0.955
**PC 11**	**4.70**	**[1.07, 8.33]**	**0.011**
**Face preference (Proportion on face)**	**Beta**	**95% CI**	**Uncorrected *p*-value**
**Corrected age (in days)**	**0.22**	**[0.13, 0.31]**	**<0.001**
Sex	-0.19	[-0.37, -0.01]	0.043
GPS autism	-0.04	[-0.16, 0.07]	0.475
GPS ADHD	-0.01	[-0.11, 0.10]	0.864
GPS bipolar disorder	-0.05	[-0.21, 0.10]	0.482
GPS major depression disorder	0.05	[-0.06, 0.15]	0.389
GPS schizophrenia	0.05	[-0.08, 0.19]	0.420
PC 2	-12.99	[-27.39, 1.40]	0.077
PC 3	-2.78	[-13.21, 7.65]	0.601
PC 4	-18.47	[-41.11, 4.17]	0.110
PC 5	-9.86	[-31.11, 11.39]	0.363
PC 6	-3.44	[-22.21, 15.33]	0.719
PC 7	-8.31	[-27.76, 11.14]	0.403
PC 8	4.66	[-13.58, 22.91]	0.616

PC 9	19.26	[-0.62, 39.14]	0.058
PC 10	-4.07	[-19.85, 22.29]	0.690
PC 11	15.04	[-2.95, 35.56]	0.161

In bold: significant predictors.

Supplementary Results 1. Analyses for the association between preference for faces and preferential looking within faces (eye versus mouth)

In this analysis we studied the etiological associations between face preference and the total eye-mouth-index (EMI), which was reported in a previous published paper. The EMI was calculated as the mean amount of gaze in the eyes AOI, divided by the mean amount of gaze to both the eyes AOI and the mouth AOI (i.e., 1 = only eyes looking; 0 = only mouth looking).

Supplementary Table 34. Descriptive statistics of the total eye-mouth-index (EMI) in the current paper sub-sample. Statistics presented as Mean (SD) / min-max. MZ, monozygotic; DZ, dizygotic.

	**Overall**	**MZ Females**	**MZ Males**	**DZ Females**	**DZ Males**	**Skewness**
**N (twins)**	492	125	144	106	117	
**Age (in days)**	167 (9)<br>145-203	168 (9)<br>149-191	167 (8)<br>150-187	167 (8)<br>153-188	167 (10)<br>145-203	0.56
**Eye-mouth-index**	.71 (.31)<br>0-1	.69 (.32)<br>0-1	.77 (.28)<br>.01-1	.69 (.31)<br>.01-1	.69 (.33)<br>0-1	-0.99

Supplementary Table 35. Assumption testing for the bivariate model between face preference and total eye-mouth-index, including covariates. The χ2 distribution and associated p-value was used to test the twin model-fitting assumptions (assumptions were violated if there was a significant decrement in fit compared to the saturated model).

					**Comparative fit with saturated model**		
**Model**	**# Parameters**	**-2LL**	***df***	**AIC**	**Δ χ2**	**Δ *df***	**P value**
Saturated	32	1637.67	996	-354.33	-	-	-
5.	15	1651.07	1013	-374.93	13.40	17	0.709

Model definitions. The Fully Sat. model is the fully saturated model of the observed data, which models the means and variances for both variables, and the phenotypic and cross-twin-cross-trait correlations between the two variables, separately for each twin in a pair and across zygosity. **5.** In the Bivariate model fitting, the constrained saturated model equates means, variances, phenotypic and cross-twin-cross-trait correlations across twins within a pair and across zygosity, for both variables of interest.
The best-fitting model (in bold) was the non-significant and most parsimonious model, as well as the one with the lowest AIC.
-2LL = fit statistic, which is minus two times the log-likelihood of the data.
df = degrees of freedom
AIC, fit statistic. Lower values denote better model fits.
Δ χ2 = difference in −2LL statistic between two models, distributed χ2.
Δ *df* = difference in degrees of freedom between two models.

Supplementary Table 36. ACE Bivariate twin model fit statistics and estimates between face preference and total eye-mouth-index. The best fitting model was selected based on non-significance (meaning that there was no decrement in fit compared to the saturated or the genetic

model, indexed by the χ2 distribution) and the AIC fit statistic (Akaike information criterion, which incorporates information about both explained variance and parsimoniousness).

Model	# Parameters	-2LL	*df*	AIC	Comparison model	Δ χ2	Δ *df*	P value	A.1	A.12	A.2	C.1	C.12	C.2	E.1	E.12	E.2
**Fully Sat**	32	1637.67	996	-354.33	NA	-	-	-									
**ACE**	15	1651.11	1013	-374.89	Fully Sat	13.44	17	0.706	.45	.01	.25	0	.26	<.01	.55	.01	.49
**AE**	**12**	**1653.15**	**1016**	**-378.85**	**ACE**	**2.04**	**3**	**0.564**	**.45 [0.33, 0.56]**	**.01 [0, 0.05]**	**.53 [0.40, 0.63]**	-	-	-	**.55 [0.44, 0.67]**	**.01 [0, 0.04]**	**.46 [0.36, 0.59]**
**CE**	12	1657.91	1016	-374.09	ACE	6.80	3	0.079	-	-	-	.36	.01	.44	.64	.01	.54
**E**	9	1738.20	1019	-299.80	ACE	87.09	6	0.000	-	-	-	-	-	-	1	.02	.98

Model definitions. The Fully Sat. model is the fully saturated model of the observed data, which models the means and variances for both variables, and the phenotypic and cross-twin-cross-trait correlations between the two variables, separately for each twin in a pair and across zygosity.
In bold: the best-fitting model was the non-significant with the lowest AIC.
-2LL = fit statistic, which is minus two times the log-likelihood of the data.
df = degrees of freedom
AIC, fit statistic. Lower values denote better model fits.
Δ χ2 = difference in −2LL statistic between two models, distributed χ2.
Δ *df* = difference in degrees of freedom between two models.
A = additive genetic influences
C = shared environment influences
E = non-shared environment influences
A-C-E.1 = variance on Phenotype 1 (face preference)
A-C-E.12 = variance on Phenotype 2 (face preference) that is shared with Phenotype 1 (EMI)
A-C-E.2 = unique variance on Phenotype 2 (EMI)

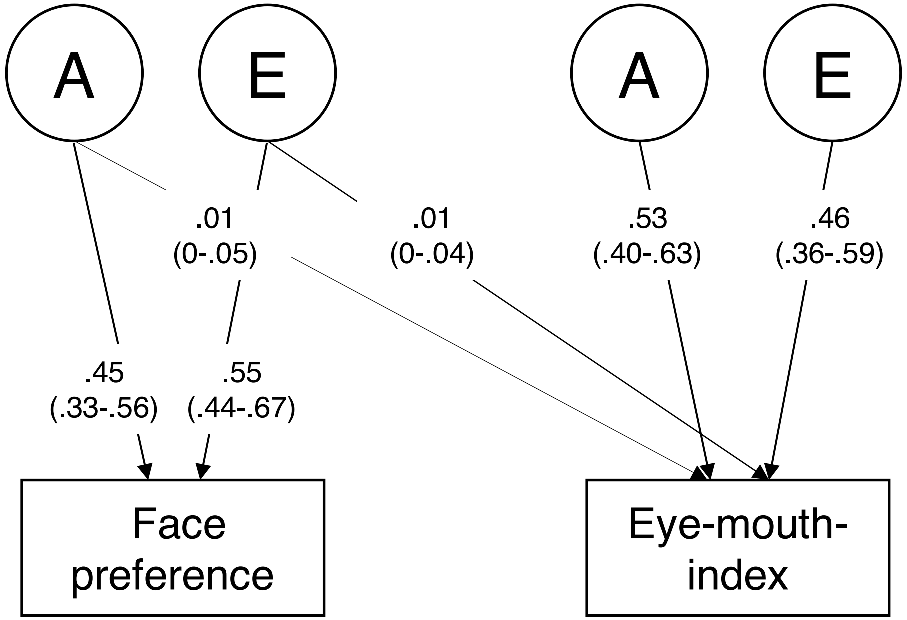

Supplementary Figure 4. ***Schematic AE bivariate twin model for face preference and eye-mouth-index.*** *A = genetic influences, E = unique environment influences.*
