# Supplementary material for: Infants’ looking preferences for social versus non-social objects reflect genetic variation
Source: Nat Hum Behav. 2023 Nov 27;8(1):115–24. doi: 10.1038/s41562-023-01764-w (PMC10810753; doi:10.1038/s41562-023-01764-w)
Supplement: Supplementary file 2 — Reporting Summary [file 41562_2023_1764_MOESM2_ESM.pdf]

nature portfolio

Corresponding author(s): Ana Maria Portugal

Last updated by author(s): Aug 25, 2023

Reporting Summary

Nature Portfolio wishes to improve the reproducibility of the work that we publish. This form provides structure for consistency and transparency in reporting. For further information on Nature Portfolio policies, see our Editorial Policies and the Editorial Policy Checklist.

Statistics

For all statistical analyses, confirm that the following items are present in the figure legend, table legend, main text, or Methods section.

n/a	Confirmed	
☐	☒	The exact sample size (*n*) for each experimental group/condition, given as a discrete number and unit of measurement
☐	☒	A statement on whether measurements were taken from distinct samples or whether the same sample was measured repeatedly
☐	☒	The statistical test(s) used AND whether they are one- or two-sided<br>*Only common tests should be described solely by name; describe more complex techniques in the Methods section.*
☐	☒	A description of all covariates tested
☐	☒	A description of any assumptions or corrections, such as tests of normality and adjustment for multiple comparisons
☐	☒	A full description of the statistical parameters including central tendency (e.g. means) or other basic estimates (e.g. regression coefficient) AND variation (e.g. standard deviation) or associated estimates of uncertainty (e.g. confidence intervals)
☐	☒	For null hypothesis testing, the test statistic (e.g. *F*, *t*, *r*) with confidence intervals, effect sizes, degrees of freedom and *P* value noted<br>*Give P values as exact values whenever suitable.*
☒	☐	For Bayesian analysis, information on the choice of priors and Markov chain Monte Carlo settings
☒	☐	For hierarchical and complex designs, identification of the appropriate level for tests and full reporting of outcomes
☐	☒	Estimates of effect sizes (e.g. Cohen's *d*, Pearson's *r*), indicating how they were calculated

Our web collection on statistics for biologists contains articles on many of the points above.

Software and code

Policy information about availability of computer code

Data collection	Gaze recording and experiment presentation was done using MATLAB (version R2013b, MathWorks, Natick, MA, USA), Psychtoolbox (version 3.0.12), and custom algorithms (part of a shared agreement and available from co-author L.M. on a reasonable request).
Data analysis	R software (version 4.0.0) was used for all data computation and analyses (the OpenMx package (version 2.18.1) was used for twin analyses and the drgee package for GEE analyses). The scripts are publicly available in OSF (https://osf.io/zseh2/?view_only=1d7a815ff87148a6af5a6b58c427419c).

For manuscripts utilizing custom algorithms or software that are central to the research but not yet described in published literature, software must be made available to editors and reviewers. We strongly encourage code deposition in a community repository (e.g. GitHub). See the Nature Portfolio guidelines for submitting code & software for further information.

Data

Policy information about availability of data

All manuscripts must include a data availability statement. This statement should provide the following information, where applicable:

- Accession codes, unique identifiers, or web links for publicly available datasets
- A description of any restrictions on data availability
- For clinical datasets or third party data, please ensure that the statement adheres to our policy

Unrestricted sharing of pseudonymized personal data was not specified in the study ethics application, hence data are not uploaded to a public repository. However, data are available from Terje Falck-Ytter (terje.falck-ytter@psyk.uu.se) on a reasonable request. Request will be responded to within 1 week. Sharing pseudonymized (coded) data from the study will require a data sharing agreement according to Swedish and EU law.

Field-specific reporting

Please select the one below that is the best fit for your research. If you are not sure, read the appropriate sections before making your selection.

☐ Life sciences ☒ Behavioural & social sciences ☐ Ecological, evolutionary & environmental sciences

For a reference copy of the document with all sections, see nature.com/documents/nr-reporting-summary-flat.pdf

Behavioural & social sciences study design

All studies must disclose on these points even when the disclosure is negative.

Study description	The study involved quantitative research methodologies. It used a classic twin design (i.e., compare similarity in monozygotic and dizygotic twin pairs) and structural equation model fitting approach to study individual differences in looking behaviours to faces vs non-face objects in infancy.
Research sample	The sample included 536 5-month-old same-sex twins (251 females, 293 monozygotic, mean age = 168 days), recruited from the greater Stockholm area in Sweden for the Babytwins Study Sweden (see Falck-Ytter et al, The Babytwins Study Sweden (BATSS): A Multi-Method Infant Twin Study of Genetic and Environmental Factors Influencing Infant Brain and Behavioral Development. Twin Res Hum Genet, 2021. 24(4): p. 217-227).
Sampling strategy	Same sex twin families living in the Stockholm area were identified via the Swedish Population Registry, and invited to participate via letters and telephone calls. In total, 1068 families were invited to join the study, of which 311 families participated in the study (n = 622 infants). The pre-established target sample size was 620 individuals (310 pairs) based on the size of previous twin studies with toddlers (e.g., Ronald et al., Exploring the relationship between autistic-like traits and ADHD behaviors in early childhood: Findings from a community twin study of 2-year-olds. Journal Of Abnormal Child Psychology, 2010. 38, 185–196) and informed by a general power calculation (see Supplementary Information). For more information about the study see Falck-Ytter et al, The Babytwins Study Sweden (BATSS): A Multi-Method Infant Twin Study of Genetic and Environmental Factors Influencing Infant Brain and Behavioral Development. Twin Res Hum Genet, 2021. 24(4): p. 217-227.
Data collection	Parents gave informed consent to take part at each time point. A gift voucher of approximately 80€ was given to each family in the first lab-assessment. Data was collected using an eye-tracking device at 5 months of age, and parent-rated on-line questionnaires at 5 months, 14 months, and 24 months. Saliva samples were also collected from infants by research assistants. The research assistants collecting the data were blind to the zygozity of the twin pairs as well as the experimental hypotheses.
Timing	The first lab-assessment (at 5 months) was collected from April 2016 to February 2020.
Data exclusions	Participants were excluded due to pre-established exclusion criteria (seizures at the time of birth, spina bifida, twin-to-twin transfusion syndrome, birthweight below 1.5 kg; n = 28 infants). Further, some infants did not complete the eye-tracking assessment due to technical reasons, time constraints, bad calibration, or tiredness (n = 23 infants), and some did not have enough valid data in the experimental task (n = 35 infants).
Non-participation	From the target population (see Recruitment section below) 29% of families ultimately participated in the lab-assessment at 5 months. At 14 months, 86% of participating families provided data for at least one questionnaire. At 24 months, 72% of families provided data for at least one questionnaire.
Randomization	Participants were not allocated into experimental groups.

Reporting for specific materials, systems and methods

We require information from authors about some types of materials, experimental systems and methods used in many studies. Here, indicate whether each material, system or method listed is relevant to your study. If you are not sure if a list item applies to your research, read the appropriate section before selecting a response.

Materials & experimental systems

n/a	Involved in the study	
☒	☐	Antibodies
☒	☐	Eukaryotic cell lines
☒	☐	Palaeontology and archaeology
☒	☐	Animals and other organisms
☐	☒	Human research participants
☒	☐	Clinical data
☒	☐	Dual use research of concern

Methods

n/a	Involved in the study	
☒	☐	ChIP-seq
☒	☐	Flow cytometry
☒	☐	MRI-based neuroimaging

Human research participants

Policy information about studies involving human research participants

Population characteristics	See above.
Recruitment	Same sex twin families living in the Stockholm area were identified via the Swedish Population Registry, and invited to participate via letters and telephone calls. 29% of the target population ultimately participated in the BATSS study. Possible self-selection sources include socio-economic status, ethnic background, and physical or mental health issues in the parents. If and how such factors affect looking preferences in infancy is not known. For more information about the sample and possible biases, see Falck-Ytter et al, The Babytwins Study Sweden (BATSS): A Multi-Method Infant Twin Study of Genetic and Environmental Factors Influencing Infant Brain and Behavioral Development. Twin Res Hum Genet, 2021. 24(4): p. 217-227.
Ethics oversight	The study was approved by the Regional Ethical Review Board in Stockholm.

Note that full information on the approval of the study protocol must also be provided in the manuscript.
